# The solid-state structure of the β-blocker metoprolol: a combined experimental and *in silico* investigation

**DOI:** 10.1107/S2053229618017084

**Published:** 2019-01-15

**Authors:** Patrizia Rossi, Paola Paoli, Laura Chelazzi, Luca Conti, Andrea Bencini

**Affiliations:** a Department of Industrial Engineering, University of Florence, Via di S. Marta 3, Florence, I-50139, Italy; b Centro di Cristallografia Strutturale, University of Florence, Via della Lastruccia 3, Sesto Fiorentino-FI, I-50019, Italy; c Department of Chemistry ‘Ugo Schiff’, University of Florence, Via della Lastruccia 3, Sesto Fiorentino-FI, I-50019, Italy

**Keywords:** metoprolol, beta-blocker, in silico, crystal structure, Hirshfeld surface, anisotropic lattice expansion

## Abstract

Metoprolol {systematic name: (*RS*)-1-iso­propyl­amino-3-[4-(2-meth­oxy­eth­yl)phen­oxy]propan-2-ol}, C_15_H_25_NO_3_, is a cardioselective β_1_-adrenergic blocking agent that shares part of its mol­ecular skeleton with a large number of other β-blockers. Results from its solid-state characterization by single-crystal and variable-temperature powder X-ray diffraction and differential scanning calorimetry are presented. Its mol­ecular and crystal arrangements have been further investigated by mol­ecular modelling, by a Cambridge Structural Database (CSD) survey and by Hirshfeld surface analysis. In the crystal, the side arm bearing the isopropyl group, which is common to other β-blockers, adopts an *all-trans* conformation, which is the most stable arrangement from modelling data. The crystal packing of metoprolol is dominated by an O—H⋯N/N⋯H—O pair of hydrogen bonds (as also confirmed by a Hirshfeld surface analysis), which gives rise to chains containing alternating *R* and *S* metoprolol mol­ecules extending along the *b* axis, supplemented by a weaker O⋯H—N/N—H⋯O pair of inter­actions. In addition, within the same stack of mol­ecules, a C—H⋯O contact, partially oriented along the *b* and *c* axes, links homochiral mol­ecules. Amongst the solid-state structures of mol­ecules structurally related to metoprolol deposited in the CSD, the β-blocker drug betaxolol shows the closest analogy in terms of three-dimensional arrangement and inter­actions. Notwithstanding their close similarity, the crystal lattices of the two drugs respond differently on increasing temperature: metoprolol expands anisotropically, while for betaxolol, an isotropic thermal expansion is observed.

## Abstract 2

The metoprolol free base has been characterized in the solid state by X-ray diffaction (both single-crystal and variable-temperature powder diffraction) and differential scanning calorimetry. These studies are supplemented by mol­ecular modelling and Hirshfeld surface analysis. Structural relationships with the strictly related betaxolol are discussed.

## Introduction   

Metoprolol, or (*RS*)-1-iso­propyl­amino-3-[4-(2-meth­oxy­eth­yl)phen­oxy]propan-2-ol (see **a** in Scheme 1), is a cardioselective β_1_-adrenergic blocking agent that has numerous medical applications, such as the treatment of acute myocardial infarction, heart failure, angina pectoris and hypertension (Benfield *et al.*, 1986 ▸; Brogden *et al.*, 1977 ▸). The drug is usually manufactured as a racemic mixture, notwithstanding the fact that the β_1_-blocking activity resides in the *S* enatiomer (Dasbiswas *et al.*, 2008 ▸). In addition, given its quite low melting point (323 K) (Ionescu *et al.*, 2006 ▸), metoprolol is always marketed in salt-based formulations (*i.e.* tartrate, succinate and fumarate) that differ in the drug-release mechanism (Wikstrand *et al.*, 2003 ▸). According to the Biopharmaceutics Classification Scheme, metoprolol belongs to the class I substances (Amidon *et al.*, 1995 ▸), meaning that it has both high aqueous solubility and intestinal permeability, which makes this API (active pharmaceutical ingredient) suitable for Extended Release (ER) formulations.

Recently, we have reported on the solid-state structure and thermal behaviour of the tartrate (Paoli *et al.*, 2016 ▸) and fumarate salts (Rossi *et al.*, 2018 ▸) studied by *in silico* and experimental techniques. In both cases, comparisons with the crystal structure of the closely related succinate salt (Bartolucci *et al.*, 2009 ▸) were made and, where possible, the results were rationalized on the basis of their respective crystal arrangements.
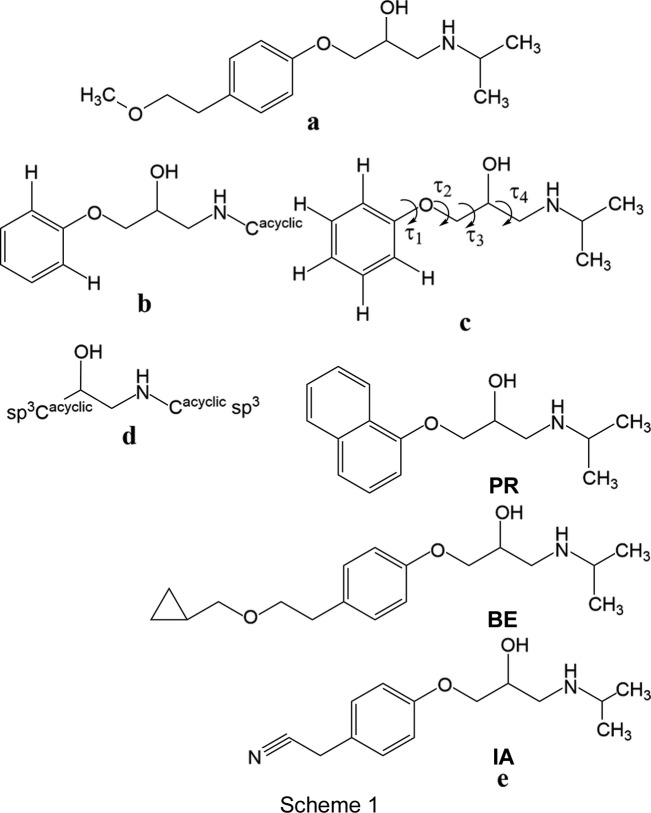


In this article, we have turned our attention to the metoprolol free base (**MB** hereafter). The inter­est in the solid-state investigation of **MB** is due to two main reasons. Firstly, metoprolol shares with a large number of β-blocker drugs the 2-hy­droxy-3-(iso­propyl­amino)­prop­oxy side arm. Therefore, it would be inter­esting to gain information about the mol­ecular structure of **MB**, in particular, the conformational preferences due to the freedom of rotation of such a side arm, and the inter­molecular inter­actions and hydrogen-bond patterns which originate from this side arm in order to find correlations between structural parameters and physicochemical properties, such as melting point and solubility (Datta & Grant, 2004 ▸), and possibly to extend these findings to closely related APIs, such as propano­lol and betaxolol. Secondly, there is a great deal of inter­est by the pharmaceutical industry in the investigation of solids containing APIs with improved physicochemical properties. In this context, the assessment of the phase stability and of the thermal behaviour of compounds of pharmaceutical inter­est (such as temperature-related phase transformations, anisotropic lattice expansion/contraction and thermal stability) provide valuable information (Rossi *et al.*, 2014 ▸; Paoli *et al.*, 2016 ▸). For example, powder X-ray diffraction (PXRD) and differential scanning calorimetry (DSC) have been used to characterize time-controlled metoprolol tartrate delivery systems using acrylic resins (Eudragit RL and Eudragit RS) for the coating. Systems containing the drug salt were compared to systems containing only the neutral metoprolol or only the tartaric acid to enable a better understanding of the inter­actions between the metoprolol salt and the film, which can strongly affect the release of the drug (Glaessl *et al.*, 2009 ▸).

Although the literature reports (Ionescu *et al.*, 2006 ▸) the analysis by single-crystal X-ray diffraction of **MB** at 173 K (**MB-173**; monoclinic crystal system, space group *P*2_1_/*n*, one independent mol­ecule in the asymmetric unit), neither the atomic coordinates nor the crystal data have been deposited in the Cambridge Structural Database (CSD; Version 5.39 of November 2017; Groom *et al.*, 2016 ▸). For this reason, the solid-state structure of metoprolol has been redetermined by a single-crystal X-ray structure analysis.

The mol­ecular structure of the metoprolol mol­ecule has been compared to those of metoprolol-like mol­ecules deposited in the CSD and the conformational space accessible to the 2-hy­droxy-3-(iso­propyl­amino)­prop­oxy side arm has been investigated by mol­ecular dynamics simulations and density functional theory (DFT) calculations.

The crystal structure has been analysed with the programs *Mercury CSD* (Macrae *et al.*, 2008 ▸) and *Crystal Explorer17* (Turner *et al.*, 2017 ▸) in order to identify the contributions to the inter­molecular contacts between the metoprolol mol­ecules and the results have been compared to those of structurally related β-blocker mol­ecules. Finally, variable-temperature PXRD (VT-PXRD) and DSC measurements were carried out in order to study thermally-induced changes, and the results are discussed.

## Experimental   

Metoprolol tartrate and betaxolol hydro­chloride were purchased from Sigma–Aldrich (product codes M5391-10G and B5683-50MG, respectively) and used without further purification.

### Synthesis and crystallization   

The metoprolol and betaxolol salts (350 and 100 mg, respectively) were dissolved in a minimal amount of Milli-Q water (0.5 and 2 ml, respectively) and passed through an anion exchange resin (Dowex Marathon 11 chloride form; Sigma–Aldrich CAS 69011-19-4) in order to obtain the free base forms of metoprolol (**MB**) and betaxolol (**BE** hereafter) directly in water. Concerning **BE**, water was completely removed by evaporation under reduced pressure and the resulting solid was dissolved in 3 ml of methanol–water (20:80%, *v*/*v*). Slow evaporation of the organic phase at low temperature (277–278 K) gave colourless crystals of **BE** suitable for single-crystal X-ray diffraction analysis after two weeks. In the case of **MB**, complete removal of the tartrate anion was confirmed by ^1^H NMR analysis of solutions of the compound in D_2_O at pD 11.10 (pH 10.70) before and after treatment with the anion-exchange resin. On purification on a column, the singlet at 4.36 ppm attributed to protons *b* and *b*′ (Qiao *et al.*, 2011 ▸) of the tartrate anion (Table S1 and Fig. S1 in the supporting information) disappears, indicating the complete absence of that anion in the final product. Removal of the solvent was performed by evaporation under reduced pressure to a final volume of *ca* 3 ml. The sample was divided into two aliquots in order to test different crystallization conditions. The first aliquot was allowed to evaporate at room temperature (298 K), resulting in the formation of a microcrystalline powder of **MB**, after 3 d, suitable for PXRD measurements. In the case of the second aliquot, a slower evaporation of the solvent, performed at 277–278 K, afforded the formation of colourless crystals of **MB** suitable for single-crystal X-ray diffraction analysis after three weeks.

### X-ray data collection and structure resolution   

The crystal structure of **MB** was investigated by means of single-crystal X-ray diffraction. Measurements were carried out at 100 K with an Rigaku Excalibur Onyx diffractometer using Cu *K*α radiation.

Crystal data, data collection and structure refinement details are summarised in Table 1 ▸. All H atoms were located from difference electron-density maps and their coordinates were refined freely, while their displacement parameters were linked to those of their parent atoms, *i.e.* *U*_iso_(H) = 1.2*U*_eq_(C,N,O), except for methyl groups, where *U*_iso_(H) = 1.5*U*_eq_(C). Table 2 ▸ lists a selection of the torsion angles. The hydrogen-bond parameters are listed in Table 3 ▸.

### Variable-temperature unit-cell parameter determination   

The crystal lattice parameters of **MB** in the 130–300 K range were determined from powder X-ray diffraction patterns measured in a vacuum using a Bruker Advance diffractometer (Cu *K*α radiation, 40 kV × 40 mA), equipped with a Bruker LYNXEYE-XE detector, scanning range 2θ = 7–35°, 0.02° increments of 2θ and a counting time of 0.8 s/step. The temperature variation rate was 10 K min^−1^ and, after the target temperature had been reached, the sample was kept for 10 min at that temperature before proceeding with data collection. The patterns underwent a Pawley fit with the software *TOPAS* (Coelho, 2012 ▸). A shifted Chebyshev polynomial with eight coefficients and a pseudo-Voigt function were used to fit the background and peak shape, respectively. The unit-cell parameters, volume and *R* factor for **MB** are summarized in Table 4 ▸. The unit-cell parameters for **BE** were determined by single-crystal diffraction analysis. Data were measured at 100, 130, 170, 210, 230, 260 and 300 K using an Rigaku Excalibur Onyx diffractometer. The unit-cell parameters are reported in Table S4 of the supporting information.

### Differential scanning calorimetry   

Differential scanning calorimetry (DSC) experiments on **MB** samples were performed on a Mettler Toledo DSC1 Excellence. Measurements were run in aluminium pans with pinhole lids (mass samples range from 1.5 to 3.5 mg). Temperature and enthalpy calibrations were done using indium as a standard. Melting point and heat of fusion (Δ*H*) were determined by measurements in the 298→343→298 K range. A linear heating rate of 10 K min^−1^ was used. Experiments were performed in air. DSC peaks were analyzed using *STAR^e^* software (Mettler–Toledo, 2018 ▸). All measurements were performed in triplicate and standard errors were ±0.1 K for temperature and ±0.3 kJ mol^−1^ for enthalpy.

### Computational methods   

Geometry optimizations (MM) and mol­ecular dynamics (MD) simulations were made using the CHARMm Force Field (Brooks *et al.*, 1983 ▸). MM calculations were performed on each species using the Smart Minimizer energy minimization procedure implemented in *Discovery Studio* (Version 2.1; Accelrys, 2018 ▸) and before starting the MD simulations the geometry of each compound was further optimized using the steepest descent and conjugate gradient algorithms. MD simulations were carried out at 100 and 300 K, both in vacuum and in an implicit water model; water calculations were performed mimicking the solvent by using a distance-dependent dielectric constant of 80. In the MD simulations, the time step was 1 fs for all runs, the equilibration time was 100 ps and the production time was 1000 ps, and snapshot conformations were sampled every 10 ps. The *Minimization*, the *Standard Dynamics Cascade* and *Analyze Trajectory*, all implemented in *Discovery Studio*, were the protocols used for energy minimization, MD simulations and analysis of MD trajectories, respectively.

*GAUSSIAN09* (Frisch *et al.*, 2010 ▸) was used for quantum chemical (QC) calculations using the following functionals: B3LYP (Becke, 1993 ▸; Stephens *et al.*, 1994 ▸) and B97-D (Grimme, 2006 ▸). The basis set was 6-311G(d,p) (Frisch *et al.*, 1984 ▸). The Berny algorithm was used (Peng *et al.*, 1996 ▸). The reliability of the stationary points was assessed by evaluation of the vibrational frequencies.

Searching on motifs (to identify inter­action motifs between mol­ecular fragments and determine their relative abundance) and *Calculating Inter­molecular Energies* using the UNI inter­molecular potentials (Gavezzotti, 1994 ▸, 1998 ▸) in order to identify the inter­molecular inter­actions which are most significant from an energetic point of view, both carried out using the CSD *Materials* software (Macrae *et al.*, 2008 ▸), were used to analyse the crystal packing arrangement.

*CrystalExplorer17* (Turner *et al.*, 2017 ▸) was used to compute Hirshfeld surfaces (HS) and their associated 2D (two-dimensional) fingerprint plots to further investigate the inter­molecular inter­actions in the crystal packing of **MB** and of the strictly related proprano­lol (**PR**), **BE** and 1-[4-(cyano­meth­yl)phen­oxy]-2-hy­droxy-3-(iso­propyl­amino)­propane (**IA**) mol­e­cules (details in Section 3.2). Total inter­action energies for a cluster of mol­ecules (mol­ecules within a radius of 3.8 Å with respect to the reference mol­ecule) of **MB** and **BE** at the B3LYP/6-31G** level of theory were also calculated. The corresponding energy frameworks were then constructed and visualized using the default values (the radii of the cylinders that make up the framework represent the relative strengths of the mol­ecular packing in different directions). In **BE**, the cyclo­propyl­meth­oxy group is disordered over two positions and the model having the highest occupancy factor was used to generate the HS and for energy calculations.

## Results and discussion   

### Mol­ecular structure from single-crystal X-ray diffraction and modelling studies   

**Figure 1:**
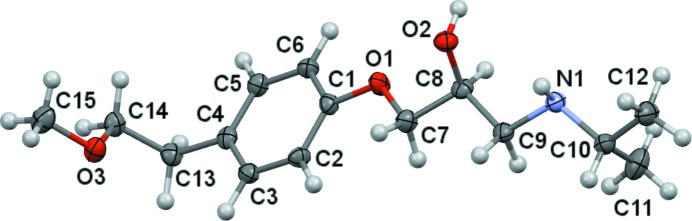
*Mercury* (Macrae *et al.*, 2008 ▸) view of the *S* enanti­omer of metoprolol in **MB** (50% probability displacement ellipsoids).

The metoprolol mol­ecule crystallizes in the monoclinic space group *P*2_1_/*n* with one mol­ecule in the asymmetric unit (Fig. 1 ▸). Because the cardiac β-blocking activity especially resides in the *S* enanti­omer, the following discussion will be focused on this isomer. Bond lengths and angles are within the expected ranges (Groom *et al.*, 2016 ▸). The side chain bearing the isopropyl group adopts an elongated conformation, with the side-chain atoms O1, C7, C8 and C9 *trans*-disposed (*all trans* or *aT*, Table 2 ▸), with all atoms, except for O2 and C11, being almost coplanar with the attached aromatic ring, as indicated by the torsion angles that define its orientation. By contrast, the 2-meth­oxy­ethyl group is perpendicularly oriented, as indicated by the value of the torsion angle about the C13—C14 bond.

**Figure 2:**
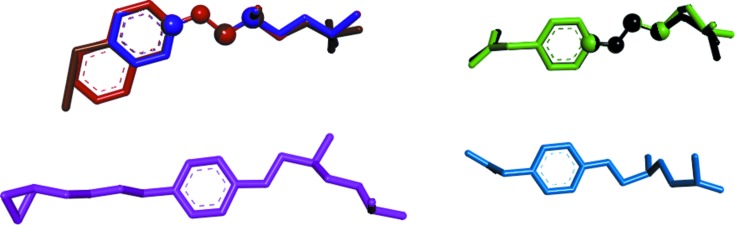
Superimposition of the X-ray structures of the neutral species found in the CSD (Groom *et al.*, 2016 ▸). Structures are superimposed as ball-and-stick atoms. H atoms have been omitted for clarity. Upper left: *aT* conformation (BEMBOK = red; GAPZEE = violet; KAZPOQ = brown); upper right: *tttg^+^* conformation (CEZVIN = black; CIDHAZ = green); lower left: *ttg^−^t* conformation (ROKNUB = pink); lower right: *ttg^+^g^+^* conformation (CIDHAZ = pale blue).

A search of the CSD was carried out to locate structures with the mol­ecular fragment sketched as **b** of Scheme 1. This moiety, which features the 2-hy­droxy-3-(iso­propyl­amino)­prop­oxy side arm together with the phenyl ring, is quite inter­esting given that it is common to a large variety of β-blocker drugs, such as atenolol, betaxolol, practolol and bis­oprolol. The CSD survey gives six compounds [neither solvated species nor salts have been taken into account; the structure of a metoprolol analogue (refcode IQEPUP; Melgar-Fernandez *et al.*, 2004 ▸) was not taken into account given its *R* configuration] which, based on the conformation adopted by the chain bearing the isopropyl group, can be classified in four different conformational families, as illustrated in Fig. 2 ▸. The superimposition of the X-ray structures of the six mol­ecules found in the CSD highlights that three of them, identified by the refcodes BEMBOK (Laguerre *et al.*, 1981 ▸), GAPZEE (Hou *et al.*, 2012 ▸) and KAZPOQ (Akisanya *et al.*, 1998 ▸), adopt the *aT* conformation (differences about the final C—N and N—C bonds have been neglected), as found in **MB** and **MB-173** (Ionescu *et al.*, 2006 ▸; see Section 1); two mol­ecules, *i.e.* CEZVIN (de Castro *et al.*, 2007 ▸) and one of the crystallographically unique mol­ecules in CIDHAZ (de Castro *et al.*, 2007 ▸), show a *trans*–*trans*–*trans*–*gauche*(+) (*tttg* ^+^) arrangement of the C—O—C—C—C atoms, while a *trans*–*trans*–*gauche*(−)–*trans* (*ttg* ^−^ *t*) conformation is shown by ROKNUB (Canotilho *et al.*,, 2008 ▸) and, finally, a *trans*–*trans*–*gauche*(+)–*gauche*(+) (*ttg* ^+^ *g* ^+^) conformation is shown by the second independent mol­ecule in the crystal packing of CIDHAZ. This conformational variability is not surprising given that side chains usually have a large conformational freedom, in addition, their conformations can be biased by inter­molecular inter­actions (*vide infra*). In this context, it appears inter­esting to study the conformational behaviour of such a chain by investigating the basic structure (**BS**, see **c** of Scheme 1) common to all the above-mentioned β-adrenoreceptor antagonists by Mol­ecular Dynamics (MD) and Quantum Chemical (QC) methods.

The **BS_aT**, **BS_TG**, **BS_GT** and **BS_GG** conformational isomers representative of the four conformational families (*aT*, *tttg* ^+^, *ttg* ^−^ *t* and *ttg* ^+^ *g* ^+^, respectively) found in the CSD were used as the starting geometries for MD simulations at 100 and 300 K, both in a vacuum and in an implicit water model.

MD trajectories collected at 100 K, in vacuum and with the implicitly simulated water medium, show overall metoprolol geometries very close to the corresponding starting rotational isomer found in the solid state (see Figs. S2, S3, S6 and S7 in the supporting information). As expected, during MD simulations at 300 K, both in vacuum and in the simulated water medium, the side arm of the metoprolol mol­ecule explores a wider portion of the conformational space. In particular, the starting geometry of the rotational isomer does not affect the space sampled, as shown by the distribution of the side-chain torsion-angle values, which is very similar irrespective of the starting geometry of metoprolol (see Figs. S4, S5, S8 and S9 in the supporting information). In particular, in all cases, τ_1_ accesses the entire range of values, τ_3_ and τ_4_ adopt a *trans* conformation in vacuum, while in simulated water, τ_4_ also populates *gauche* conformations. By contrast, τ_2_ appears frozen in the starting *trans* conformation both in vacuum and simulated solvent, but in vacuum, at least 85% of the snapshot conformations feature an O—H⋯O intra­molecular contact (distance less than 2.5 Å), while in simulated solvent, the percentage drops to 18%. Similarly, intra­molecular N—H⋯O contacts (distance less that 2.5 Å) are observed in at least 86% of the sampled conformations in vacuum, while the inclusion of a distance-dependent dielectric constant makes such an inter­action definitely less important (it is present in less than 24% of the snapshot conformations).

From each MD trajectory at 300 K both in vacuum and in the implicitly simulated water medium, ten snapshot conformations were extracted and their geometries optimized; the *all-trans* rotational isomer, *i.e.* the same as found in the solid state of metoprolol, is always the most stable. An identical result comes from QC geometry optimization: the **BS_aT** conformational isomer which, as expected, features O—H⋯O and N—H⋯O contacts, has the lowest energy content, while the **BS_GG** isomer is the highest in energy [Δ*G*_298_ = 13.19 (B3LYP) and 5.19 kJ mol^−1^ (B97-D)].

In summary, the amino­hydroxy side arm appears quite flexible, being able to change its 3D arrangement in response to the environment, as provided by the X-ray (crystal environment), MD (in vacuum and simulated solvent) and QC (in vacuum) data. Modelling results identify the *all-trans* conformation as the most stable, irrespective of the model (MM *versus* QC) and of the medium (vacuum *versus* simulated solvent), which, consistently, is the most populated in the solid state (X-ray data of **MB/MB-173**, BEMBOK, GAPZEE and KAZPOQ).

### Crystal structure from single-crystal X-ray diffraction and computational studies   

**Figure 3:**
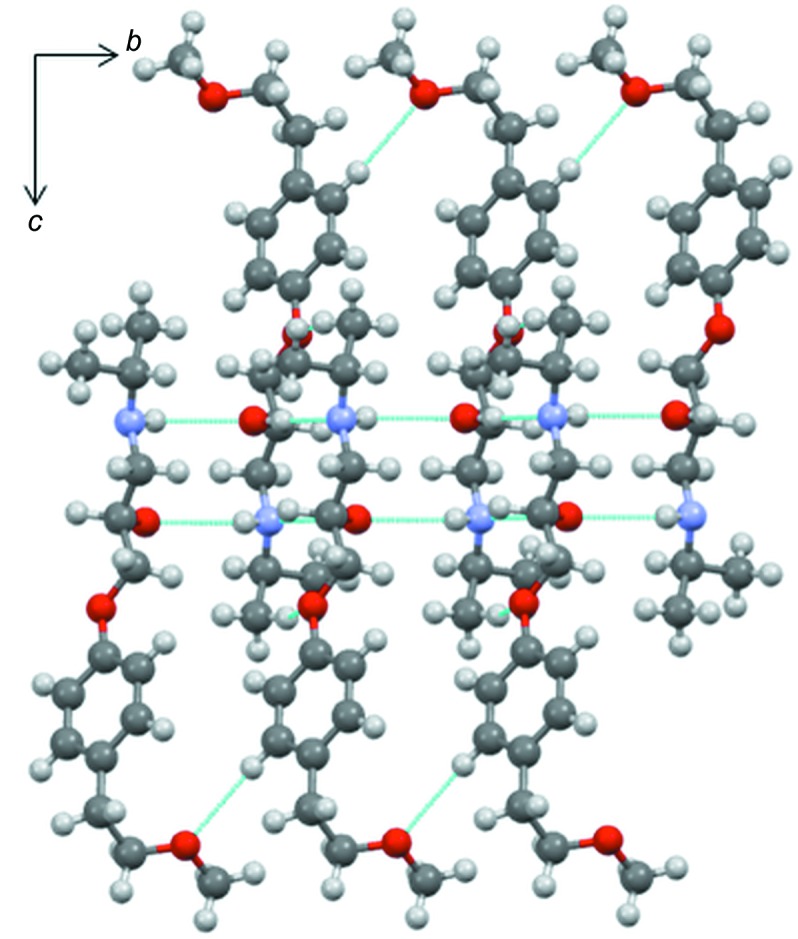
View along the *a*-axis direction of the zigzag chains of **MB** propagating parallel to the *b* axis.

In the crystal lattice, alternating *R* and *S* mol­ecules of metoprolol related by an inversion centre give rise to zigzag chains extending along the *b* axis. A view of the crystal packing along the *a* axis is presented in Fig. 3 ▸. As already reported by Ionescu *et al.* (2006 ▸), within the chain, each mol­ecule is held in place by two pairs of inter­molecular hydrogen bonds involving the hy­droxy and amine groups, which both act as hydrogen-bond donors and acceptors (Table 3 ▸). For symmetry reasons, each pair of hydrogen bonds consists of two identical inversion-related O—H⋯N/N⋯H—O and O⋯H—N/N—H⋯O hydrogen bonds. When the hy­droxy group acts as a donor toward the N atom of an inversion-related mol­ecule, the resulting hydrogen bond is strong (Desiraju & Steiner, 1999 ▸) [O2—H2⋯N1^i^ = 1.92 (3) Å and 178 (3)°; symmetry code: (i) −*x* + 1, −*y* + 2, −*z* + 1; Table 3 ▸]; by contrast, the hy­droxy group acts as a definitely weaker hydrogen-bond acceptor toward the N—H group of an inverted neighbouring mol­ecule [N1—H1⋯O2^ii^ = 2.39 (3) Å and 142 (2)°; symmetry code: (ii) −*x* + 1, −*y* + 1, −*z* + 1; Table 3 ▸]. As a whole, these inter­actions give raise to two intrachain hydrogen-bond patterns of 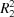
(10) type [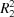
(10)>*a*>*a* and 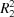
(10)>*b*>*b*] (Bernstein *et al.*, 1995 ▸), which are responsible for the formation of infinite chains of metoprolol mol­ecules extending along the *b*-axis direction (Fig. S10 of the supporting information). Finally, a relatively weak inter­action of the C—H⋯O type [C5—H5⋯O3^iii^ = 2.56 (3) Å and 160 (2)°; symmetry code: (iii) *x*, *y* + 1, *z*; Fig. 3 ▸] partially oriented along the *b* and *c* axes, exists between homochiral mol­ecules belonging to the same chain.

**Figure 4:**
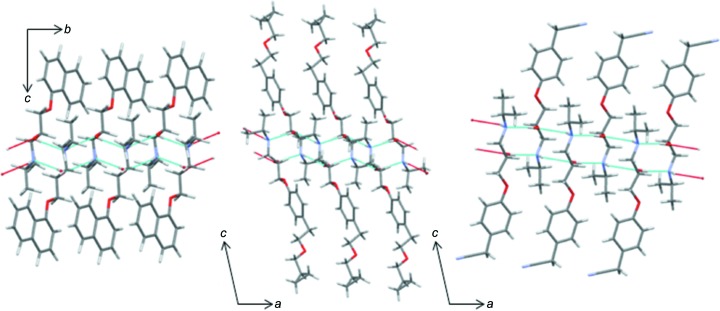
Views of the crystal lattices of (left) propranolol (**PR**), (centre) betaxolol (**BE**) and (right) a precursor of atenolol (**IA**), showing chains of mol­ecules propagating along the shortest axis direction describing an 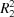
(10) hydrogen-bond pattern.

Since the most significant hydrogen-bond motif, *i.e.* 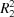
(10) involves the mol­ecular fragment that metropolol shares with a large number of β-blocker drugs, the CSD was searched to find which hydrogen-bond motifs are formed most commonly by a pair of the mol­ecular fragments sketched as **d** of Scheme 1 through O—H⋯N(—H) inter­actions and the occurrence of the double 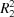
(10) motif. In most of the retrieved hits (70.6%), at least one O—H⋯N(—H) inter­action holds the two mol­ecular fragments together. The most common motifs are an infinite chain (*C*1, *i.e.* chain, one contact), with frequency 29.4% (calculated as the number of hits found/number of structures that feature the searched fragment), followed by *R*2 (*i.e.* ring, two contacts) (27.5%), while rings with four contacts (*R*4) represent about 10% of the sample (these motif descriptors are not the same as graph-set notation). Three (see **e** of Scheme 1) of the 14 structures featuring an *R*2 pattern show the same motif [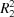
(10)>*a*>*a* and 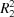
(10)>*b*>*b*] as found in **MB** (and **MB-173**): two of them, propranolol [refcode PROPRA10 (Ammon *et al.*, 1977 ▸), **PR** in the following] and betaxolol (ROKNUB, **BE** in the following) belong to the β-blocker class of drugs, the third is a reaction inter­mediate in an alternative route for the synthesis of atenolol (KAZPOQ, **IA** in the following). As already found for **MB**, in all three cases, mutually inverted mol­ecules face each other and are held together by hydrogen bonds between the hy­droxy and amino groups, giving rise to chains extending along the shortest axis direction (Fig. 4 ▸ and Table S2 in the supporting information). In other words, the number, types, geometry and patterns of the inter­molecular hydrogen bonds described by the OH/NH groups are practically identical. Thus, the overall packing arrangements, as well as the densities and the Kitaigorodskii packing index (KPI) (Kitaigorodskii, 1961 ▸; Spek, 1998 ▸), are very similar (Table S3 in the supporting information).

As already found for **MB**, and also in **PR**, **BE** and **IA**, hydrogen bonds are definitely stronger when OH acts as a donor than when it acts as an acceptor. Accordingly, in all the crystal lattices, the most significant inter­action in energetic terms, as suggested by the inter­molecular potential calculated using the empirical UNI pair potential parameters (Gavezzotti 1994 ▸, 1998 ▸), is between the pair of mol­ecules held together by the O—H⋯N/N⋯H—O pair of hydrogen bonds. The O⋯H—N/N—H⋯O pair of inter­actions appears less important from an energetic point of view and, in **PR** and **BE**, it even ranks third among the strongest inter­actions (second in **MB** and **IA**; Fig. S11 in the supporting information). In **PR** and **BE**, the relative arrangement of the pair of mol­ecules involved in the second strongest inter­action (Fig. S12 in the supporting information) suggests that π–π parallel-displaced inter­actions in **PR** (along *b*) and C—H⋯π contacts in **BE** (along *a*) are at work within each chain (for geometrical details, see Table S2 in the supporting information).

**Figure 5:**
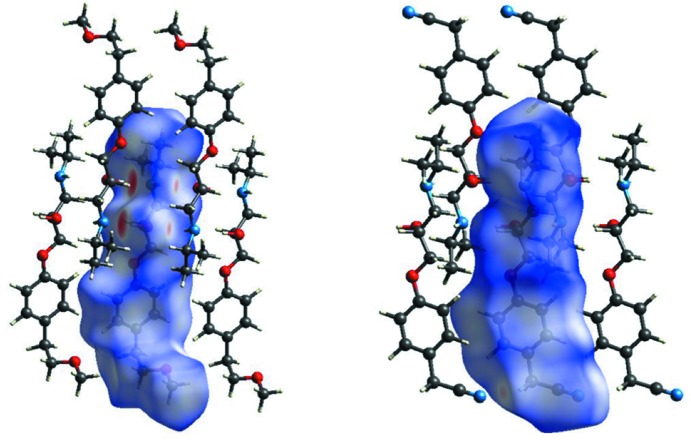
*d*_norm_ surfaces of **MB** (left) and **AI** (right). Neighbouring mol­ecules associated with close contacts are also shown.

The inter­molecular inter­actions which hold together **MB**, **IA**, **PR** and **BE** in their respective solids were further investigated using Hirshfeld surface (HS) analysis. The corresponding HSs mapped with *d*_norm_ highlighting the inter­molecular contacts are shown in Figs. 5 ▸ and 6 ▸; in all cases, the dominant inter­action is the O—H⋯N/N⋯H—O pair of hydrogen bonds (two large red spots); the weaker O⋯H—N/N—H⋯O couple of inter­actions, as well as less prominent contacts, show up as pale-red regions. The corresponding fingerprint plots are given in Fig. S13 in the supporting information. All the fingerprint plots feature a pair of spikes which represent the hydrogen bonds involving the NH/OH groups (upper left OH donor, bottom left NH acceptor) and two well-defined lateral wings (except **PR**, see later) which account for C—H⋯π contacts. Finally Fig. S14 in the supporting information shows the fingerprint plots broken down into contributions from N⋯H and C⋯C close contacts for the four mol­ecules presented here (Fig. S15 shows the other contributions). From these data, it emerges that the nature and contribution of the inter­molecular contacts of **MB** and **BE**, which differ with respect to the terminal group (isopropyl instead of cyclo­prop­yl), are very similar; thus, they have almost identical roles in the corresponding crystal packing. By contrast, in **PR** and **IA**, C⋯C and N⋯H contacts also contribute to the crystal packing through the naphthalene group in **PR** and the cyano group in **IA**.

**Figure 7:**
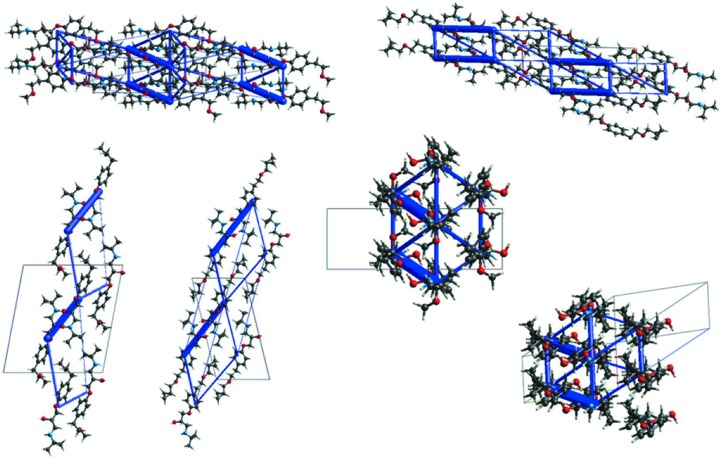
Energy frameworks corresponding to the total inter­action energy in **MB** and **BE** (views are chosen in order to highlight similarities between **MB** and **BE**).

Due to the close similarity between **MB** and **BE**, the following discussion focuses on these two compounds. Results from inter­molecular inter­action energy calculations (B3LYP and HF energy models) between mol­ecular pairs in **MB** and **BE** confirm that the O—H⋯N/N⋯H—O pair of hydrogen bonds are by far the most important inter­actions, followed by the C—H⋯π inter­actions in **BE**, while in **MB**, all the other contacts are almost isoenergetic and definitely less important from an energetic point of view. The values of the inter­action energy calculated between the closest mol­ecules are used to construct the energy framework shown in Fig. 7 ▸. A comparison of the total energy frameworks evidences the strict similarity between **MB** and **BE**. For example, the views along the *a* and *b* axes of **MB** look very similar to the views down the *b* and *a* axes of **BE** (the same applies when viewing down the mol­ecular axis).

In conclusion, an analysis of the inter­molecular contacts in **MB**, in terms of geometry, motifs, Hirshfeld surface and inter­molecular energies, highlights the close resemblance of the metoprolol crystal packing with that of another β-blocker drug betaxolol. In both cases, the O—H⋯N/N⋯H—O hydrogen bonds appear to drive the arrangement of the mol­ecules in the corresponding solid, giving rise to chains of alternating *R* and *S* mol­ecules which propagate along the shortest axis direction.

### Crystal structure from X-ray microcrystalline powder diffraction and differential scanning calorimetry analysis   

The correspondence between the crystal structure of metoprolol free base, as determined by single-crystal X-ray diffraction (**MB**), and that of the bulk material was checked by comparing calculated (150 K) and measured (130 K) powder diffraction patterns (Fig. S16 in the supporting information). DSC measurements performed in the 298–343 K range do not show any evidence of a thermal event (see Fig. S17 in the supporting information), except that related to the melting at around 324 K (peak 323.9 K, extrapolated peak 324.0 K) with a melting enthalpy of 188.1 J g^−1^ (50.3 kJ mol^−1^). Consistently, the XRPD patterns measured in the temperature range 130–300 K superimpose quite well (Fig. S18 in the supporting information); there were no differences in the overall number of peaks and in their relative intensities on heating. Thus, no phase changes occur under these experimental conditions up to the **MB** melting point. However, a closer inspection shows that peaks shift to a different extent as the temperature is increased; several peaks move towards lower 2θ values, while the position of others remains almost unchanged, thus suggesting that an anisotropic thermal expansion takes place on raising the temperature. In particular, the shift is evident for the (*h*00) and (*h*0*l*) peaks; by contrast, the (0*k*0), (*hk*0) and (0*kl*) peaks do not shift significantly with the increasing temperature. The knowledge that **MB** undergoes an anisotropic expansion on heating could be relevant when the phase purity of the API, as well as its phase composition in formulations, is checked by a comparison of the powder diffraction pattern of a sample with a reference powder pattern: unexpected differences due to anisotropic lattice expansion/contraction could lead to wrong conclusions about phase purity/composition. In particular, the lattice parameters calculated from the XRPD patterns (see Section 2) listed in Table 4 ▸ confirm this observation; the *a* axis expands significantly with respect to both *b* (which by contrast slightly contracts) and *c* (which remains almost unchanged). This trend is well qu­anti­fied by the linear thermal expansion coefficients (TECs; Hori *et al.*, 2007 ▸; Krishnan *et al.*, 1979 ▸) listed in Table 5 ▸ (and it is only partially accounted for by the inter­molecular hydrogen bonds involving the hy­droxy and amine groups that link the metoprolol mol­ecules along the *b* axis).

Due to the crystal packing similarities between **MB** and **BE**, it appears inter­esting to assess the thermal behaviour of betaxolol. In the investigated temperature range (*i.e.* 100–300 K), **BE** does not undergo any phase transition, as shown by the single-crystal X-ray diffraction data. Polymorph I of betaxolol (Maria *et al.*, 2013 ▸) is stable under the experimental conditions adopted and, what is more, the crystal lattice expands isotropically on raising the temperature (the linear thermal expansion coefficient for **BE** is reported in Table S5 of the supporting information).

Thus, notwithstanding the strict similiarity between the crystal packings of **MB** and **BE** in terms of the nature of the most significant inter­molecular contacts (number, type, geometry, motifs, inter­action energies) and packing efficiency (density, KPI), they respond differently to thermal stimulus; the metoprolol lattice expands anisotropically, while by contrast and quite surprisingly, an isotropic expansion is observed for betaxolol. Besides, the latter shows, as reported by Canotilho (Canotilho *et al.*, 2008 ▸), a slightly higher melting temperature, 341 K, but a smaller melting enthalpy (45.9 kJ mol^−1^). In other words, crystal structure similarities do not imply similar macroscopic properties.

Therefore, however much has been done within the framework of structure–property/function relationships, much remains to be done, especially when APIs are concerned, due to the relevance of their solid-form properties in view of their pharmaceutical development.

## Supplementary Material

Crystal structure: contains datablock(s) I. DOI: 10.1107/S2053229618017084/ky3155sup1.cif

Structure factors: contains datablock(s) I. DOI: 10.1107/S2053229618017084/ky3155Isup2.hkl

Supporting information file. DOI: 10.1107/S2053229618017084/ky3155Isup3.cml

PDF document (Supporting material: Additional H-bond interactions table and figure, additional crystal data tables, 1H-NMR spectrum and signal attribution, additional XRPD patterns, DSC curve, fingerprint plots, intermolecular potentials, dihedral angles distribution from MD simulation.). DOI: 10.1107/S2053229618017084/ky3155sup4.pdf

CCDC reference: 1882466

## Figures and Tables

**Figure 6:**
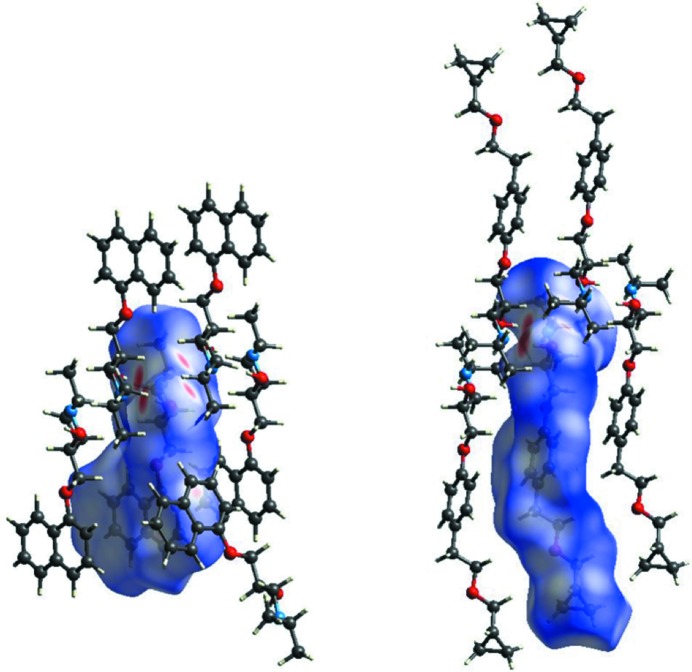
*d*_norm_ surfaces of **PR** (left) and **BE** (right). Neighbouring mol­ecules associated with close contacts are also shown.

**Table 1:** Experimental details

Crystal data	
Chemical formula	C_15_H_25_NO_3_
*M*_r_	267.36
Crystal system, space group	Monoclinic, *P*2_1_/*n*
Temperature (K)	100
*a*, *b*, *c* (Å)	16.0344 (3), 5.4375 (1), 17.8512 (3)
β (°)	100.731 (2)
*V* (Å^3^)	1529.18 (5)
*Z*	4
Radiation type	Cu *K*α
μ (mm^−1^)	0.64
Crystal size (mm)	0.25 × 0.20 × 0.14

Data collection	
Diffractometer	Rigaku Excalibur Onyx
Absorption correction	Multi-scan (*CrysAlis PRO*; Rigaku OD, 2018 ▸)
*T*_min_, *T*_max_	0.923, 1.000
No. of measured, independent and observed [*I* > 2σ(*I*)] reflections	6999, 2915, 2070
*R*_int_	0.059
(sin θ/λ)_max_ (Å^−1^)	0.618

Refinement	
*R*[*F* ^2^ > 2σ(*F* ^2^)], *wR*(*F* ^2^), *S*	0.050, 0.121, 1.04
No. of reflections	2915
No. of parameters	247
H-atom treatment	Only H-atom coordinates refined
Δρ_max_, Δρ_min_ (e Å^−3^)	0.23, −0.22

Computer programs: *CrysAlis PRO* (Rigaku OD, 2018 ▸), *SIR2004* (Burla *et al.*, 2005 ▸), *SHELXL2014* (Sheldrick, 2015 ▸), *ORTEP-3* (Farrugia, 2012 ▸), *Mercury* (Macrae *et al.*, 2008 ▸), *Discovery Studio Visualizer* (Accelrys, 2018 ▸) and *PARST97* (Nardelli, 1995 ▸).

**Table 2:** Selected torsion angles (°) (*S* enanti­omer) for **MB** and **MB-173**

Torsion angle	**MB**	**MB-173** ^*a*^
C2—C1—O1—C7	4.7 (3)	4.5
C1—O1—C7—C8	177.8 (2)	177.6
O1—C7—C8—C9	−162.4 (2)	−162.9
C7–C8—C9—N1	−176.0 (2)	−176.0
C8—C9—N1—C10	−163.4 (2)	−163.4
C9—N1—C10—C11	76.0 (2)	76.7
C9—N1—C10—C12	−161.4 (2)	−161.2
C3—C4—C13—C14	97.3 (2)	98.1
C4—C13—C14—O3	−74.1 (2)	−73.2
C13—C14—O3—C15	177.4 (2)	177.6

Reference: (*a*) Ionescu *et al.* (2006 ▸).

**Table 3:** Hydrogen-bond geometry (Å, °)

*D*—H⋯*A*	*D*—H	H⋯*A*	*D*⋯*A*	*D*—H⋯*A*
O2—H2O⋯N1^i^	0.89 (3)	1.92 (3)	2.808 (3)	178 (3)
N1–H1N⋯O2^ii^	0.93 (3)	2.39 (3)	3.167 (3)	142 (2)
C5—H5⋯O3^iii^	1.01 (3)	2.56 (3)	3.526 (3)	160 (2)
C13—H13*A*⋯O3^iii^	1.011 (3)	2.910 (4)	3.730 (4)	138.7 (2)
C15—H15*A*⋯O3^iv^	0.934 (4)	3.11 (1)	3.93 (1)	147 (3)

Symmetry codes: (i) −*x* + 1, −*y* + 2, −*z* + 1; (ii) −*x* + 1, −*y* + 1, −*z* + 1; (iii) *x*, *y* + 1, *z*; (iv) −*x* + 1, −*y* + 1, −*z*.

**Table 4:** Unit-cell parameters, volume (*V*) and *R* factor for **MB** at different temperatures from XRPD data

*T* (K)	*a* (Å)	*b* (Å)	*c* (Å)	β (°)	*V* (Å^3^)	*R*_wp_
130	16.103 (2)	5.459 (1)	17.858 (6)	100.588 (7)	1543.1 (6)	5.77
170	16.202 (1)	5.4581 (8)	17.865 (3)	100.521 (7)	1553.3 (4)	5.87
190	16.258 (3)	5.453 (1)	17.862 (6)	100.471 (8)	1557.3 (6)	5.75
230	16.3816 (7)	5.4477 (6)	17.875 (2)	100.408 (7)	1568.9 (3)	6.27
260	16.4789 (6)	5.4394 (6)	17.892 (2)	100.349 (6)	1577.6 (3)	5.92
300	16.5703 (9)	5.4259 (8)	17.889 (8)	100.226 (8)	1582.8 (3)	6.32

**Table 5:** Linear (α) and volume (β) thermal expansion coefficients (TECs) calculated for **MB** taking as reference the unit-cell parameter values calculated at 130 K

*T* (K)	α_*a*_ (10^−5^) C^−1^	α_*b*_ (10^−5^) C^−1^	α_*c*_ (10^−5^) C^−1^	β (10^−4^) C^−1^
130	–	–	–	–
170	15.4	−0.4	1.0	1.6
190	16.0	−1.8	0.4	1.5
230	17.3	−2.1	0.9	1.7
260	18.0	−2.8	1.5	1.7
300	17.1	−3.6	1.0	1.5
