# Supplementary material for: The solid-state structure of the β-blocker metoprolol: a combined experimental and in silico investigation
Source: Acta Crystallogr C Struct Chem. 2019 Jan 15;75(Pt 2):87–96. doi: 10.1107/S2053229618017084 (PMC6363042; doi:10.1107/S2053229618017084)
Supplement: Supplementary file 4 [file c-75-00087-sup4.pdf]

The solid state structure of the ß-blocker metoprolol: a combined experimental and in silico investigation

Patrizia Rossi,[a] Paola Paoli,[a*] Laura Chelazzi,[b] Luca Conti[c] and Andrea Bencini[c]

[a] Dept. of Industrial Engineering, University of Florence, Via di S. Marta 3, I-50139 Florence, Italy, [b]Centro di Cristallografia Strutturale, University of Florence, Via della Lastruccia 3, I-50019 Sesto Fiorentino-FI, Italy, [b]Dept. of Chemistry "Ugo Schiff", University of Florence, Via della Lastruccia 3, I-50019 Sesto Fiorentino-FI, Italy

Supplementary Material

Table S1: Signal attribution (Qiao *et al.*, 2011) used for the ^{1}H-NMR analysis of metoprolol tartrate in D_2O at pD 11.10.

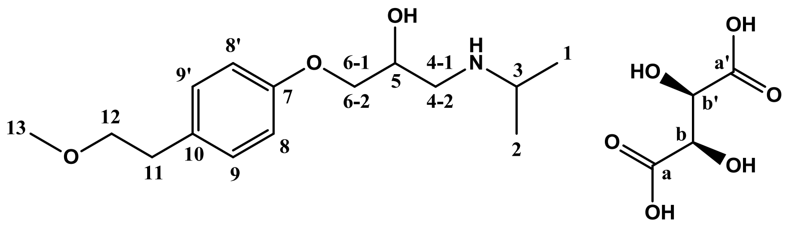

δ 7.29 ppm, d (2H), 9-H, 9’-H;

δ 7.02 ppm, d (2H), 8-H, 8-H’;

δ 4.36 ppm, s (2H), b-H,b’-H;

δ 4.14 ppm, m (2H), 6(1)-CH_2, 6(2)-CH_2;

δ 4.04 ppm, m (1H), 5-H;

δ 3.74 ppm, t (2H), 12-CH_2;

δ 3.37 ppm, s (3H), 13-CH_3;

δ 2.88 ppm, m (4H), 4(1)-CH_2, 4(2)-CH_2,11-CH_2;

δ 2.77 ppm, m (1H), 3-H;

δ 1.11 ppm, dd (6H), 1-CH_3, 2-CH_3;

Table S2: Intermolecular H-bonds and contacts in compound **PR** [a] **BE**[b] and **AI**[c].

	Intermolecular H-bonds			
		D···A (Å)	H···A (Å)	X-H···A (°)
**PR**	OH···N	2.81	2.10	151
	NH···O	3.17	2.44	146
**BE**	OH···N	2.86	2.04	174
	NH···O	3.38	2.56	161
**AI**	OH···N	2.84	1.93	172
	NH···O	3.21	2.49	141
	Intermolecular contacts			
		X···X[d] (Å)		
**PR**	π-π	3.755		
		H···X[e] (Å)		
**BE**	CH··· π	3.176		

[a]: refcode PROPRA10 in the Cambridge Structural Database
[b]: refcode ROKNUB in the Cambridge Structural Database
[c]: refcode KAZPOQ in the Cambridge Structural Database
[d]: X is the centroid of the facing aromatic rings
[e]: X is the centroid of the aromatic ring involved in the contact

Table S3: Crystallographic data for **PR** [a] **BE**[b] and **AI**[c].

	**PR**	**BE**	**AI**
Crystal system, space group	Monoclinic, $P2_1/c$	P-1	P-1
Unit cell dimensions (Å, °)	a = 11.7599(18)<br>b = 4.8068(6)<br>c = 26.5086(27)<br>β = 99.89(2)	a = 4.9799(11)<br>b = 10.010(2)<br>c = 19.123(3)<br>α = 103.022(17)<br>β = 91.29(3)<br>γ = 102.079(16)	a = 5.4490(4)<br>b = 8.0200(4)<br>c = 16.2340(5)<br>α = 95.2510(10)<br>β = 96.5730 (10)<br>γ = 94.9120(10)
Z, D_c (mg/cm^3)	1.17	1.127	1.18
KPI[d]	0.69	0.65	0.66

[a]: refcode PROPRA10 in the Cambridge Structural Database
[b]: refcode ROKNUB in the Cambridge Structural Database
[c]: refcode KAZPOQ in the Cambridge Structural Database
[d]: calculated by using PLATON, A Multipurpose Crystallographic Tool; Spek, A.L., Utrecht University: Utrecht, The Netherlands, 1998.

Table S4. Cell parameters and Volume for **BE** at different temperature from single crystal diffraction data.

T (K)	a (Å)	b (Å)	c (Å)	α (°)	β (°)	γ (°)	V (Å^3)
100	4.9388(5)	9.802(1)	18.915(2)	102.924(9)	93.016(1)	101.903(9)	868.6(1)
130	4.9406(7)	9.818(1)	18.935(2)	102.81(1)	92.76(1)	101.92(1)	872.1(2)
170	4.9462(8)	9.844(1)	18.976(3)	102.75(1)	92.23(1)	101.95(1)	878.2(1)
210	4.9539(8)	9.884(1)	19.015(2)	102.79(1)	92.01(1)	102.00(1)	884.9(2)
230	4.9571(9)	9.910(2)	18.999(5)	102.69(2)	91.85(2)	101.95(2)	887.7(3)
260	4.9648(7)	9.943(2)	19.059(3)	102.93(1)	91.63(1)	102.00(1)	894.1(3)
300	4.9692(9)	10.000(1)	19.122(3)	103.01(1)	91.22(1)	102.00(1)	903.3(3)

Table S5. Linear (α) and volume (β) thermal expansion coefficients (TECs) calculated for **BE** taking as reference the cell parameter values calculated at 100K.

T(K)	α a(10^{-5})C^{-1}	α b(10^{-5})C^{-1}	α c(10^{-5})C^{-1}	β (10^{-4})C^{-1}
100K				
130	1.2	5.4	3.5	1.3
170	2.1	6.1	4.6	1.6
210	2.8	7.6	4.8	1.7
230	2.8	8.5	3.4	1.7
260	3.3	9.0	4.8	1.8
300	3.1	10.1	5.5	2.0

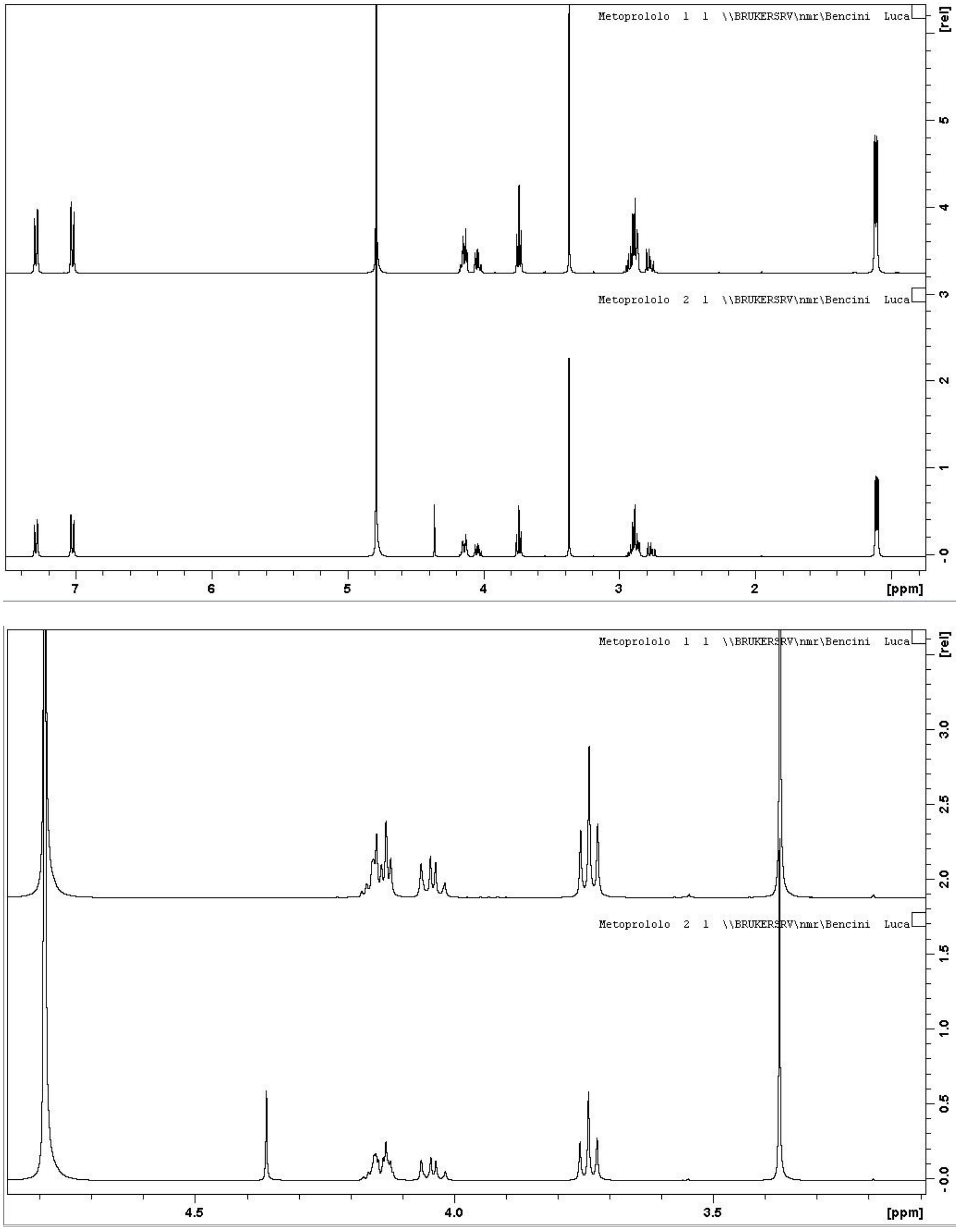

Figure S1: *a*) ^{1}H-NMR analysis of Metoprolol tartrate in D_2O a pD 11.10 before and after treatment with anion exchange resin. *b*) Magnification of the aliphatic region of the above reported spectra.

Dihedral angles (τ_1-τ_4) distribution during MD simulations at 100K in vacuum.

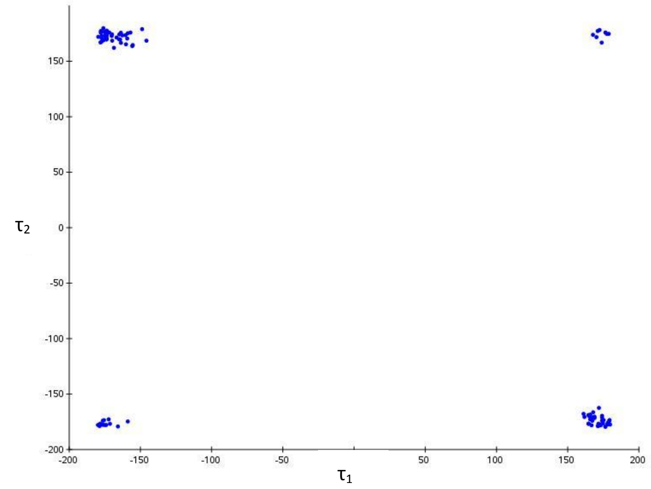

τ_1-τ_2 , *starting conformation: all trans*

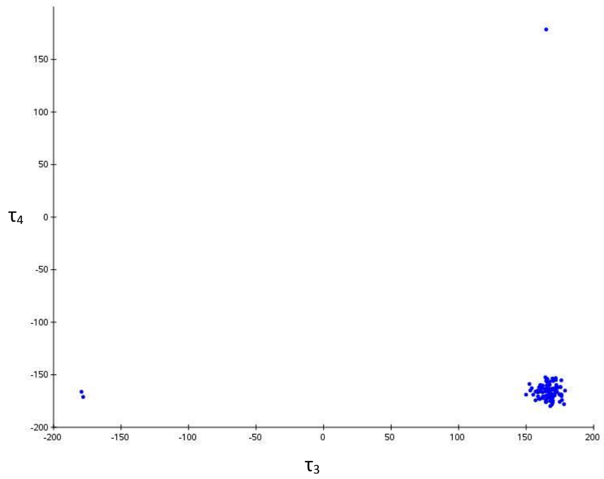

τ_3-τ_4 , *starting conformation: all trans*

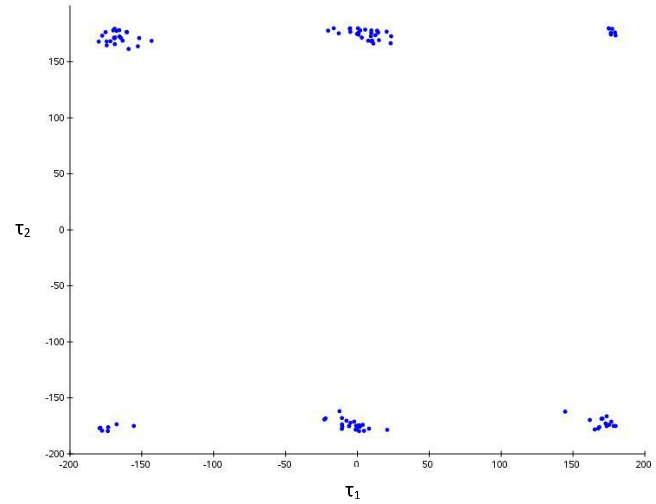

τ_1-τ_2 , *starting conformation: tttg*$^+$

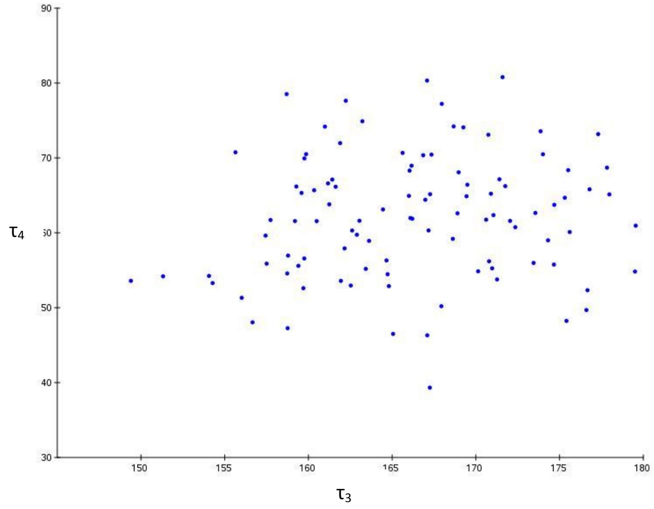

τ_3-τ_4 , *starting conformation: tttg*$^+$

Figure S2

Dihedral angles (τ_1-τ_4) distribution during MD simulations at 100K in vacuum.

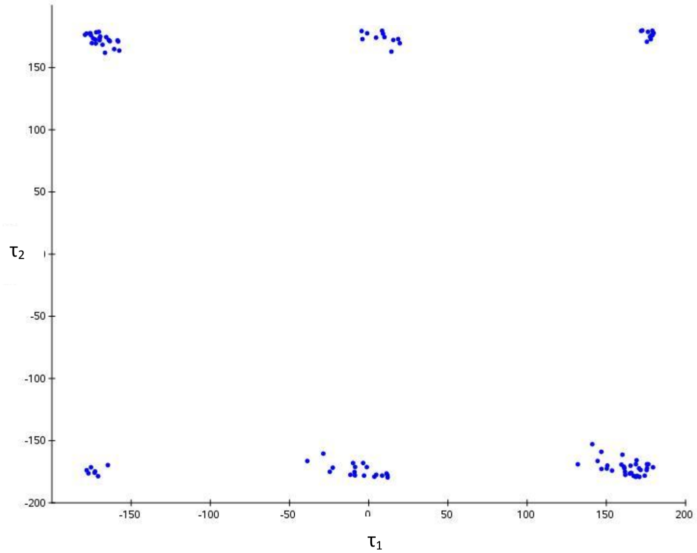

τ_1-τ_2 , *starting conformation: tt g^+g^+*

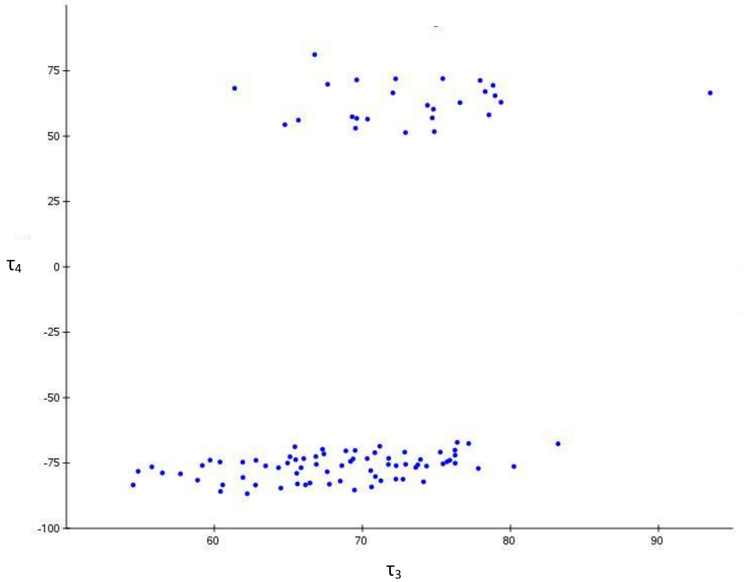

τ_3-τ_4 , *starting conformation: tt g^+g^+*

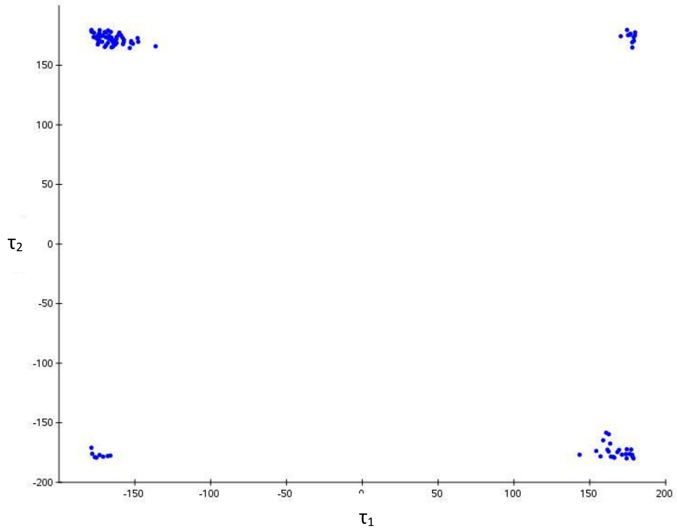

τ_1-τ_2 , *starting conformation: tt g^-t*

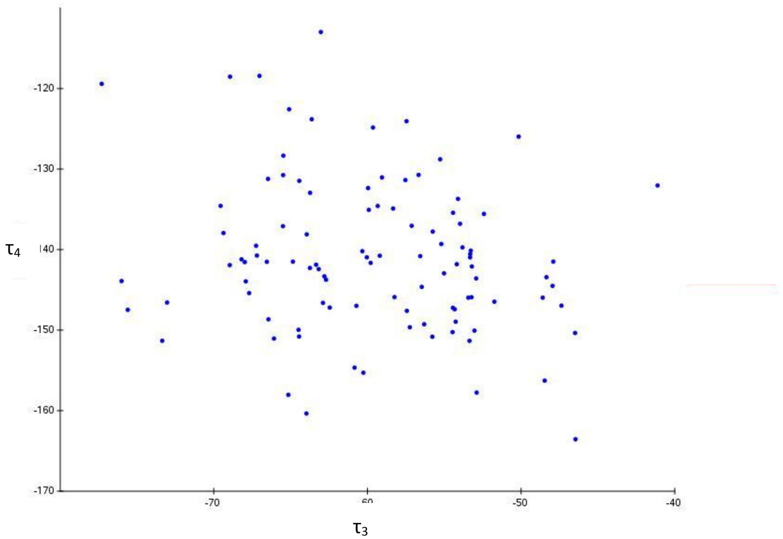

τ_3-τ_4 , *starting conformation: tt g^-t*

Figure S3

Dihedral angles (τ_1-τ_4) distribution during MD simulations at 300K in vacuum.

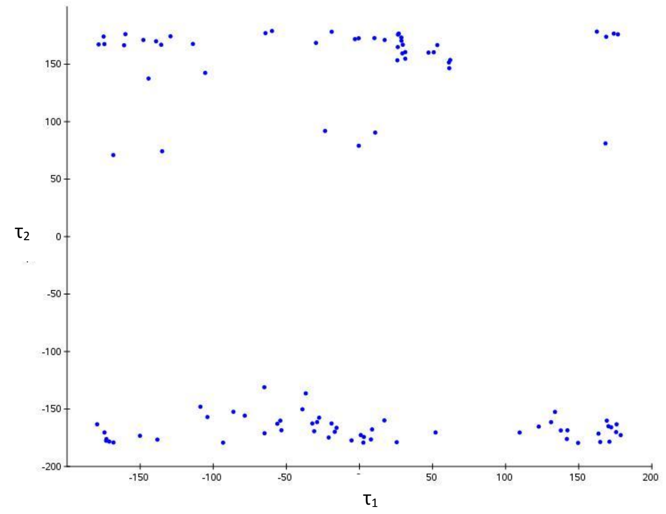

τ_1-τ_2 , *starting conformation: all trans*

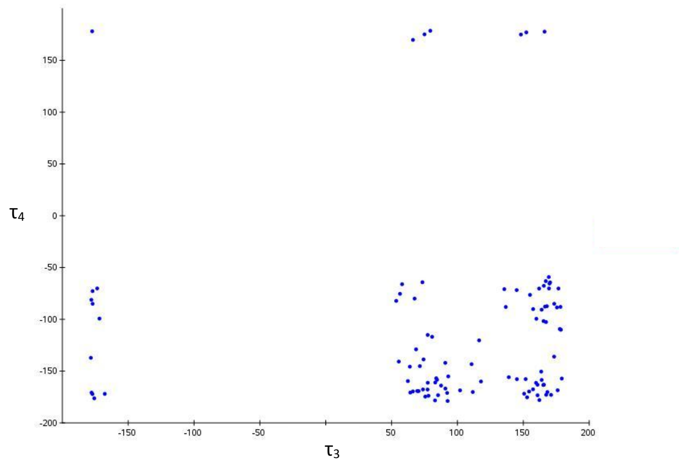

τ_3-τ_4 , *starting conformation: all trans*

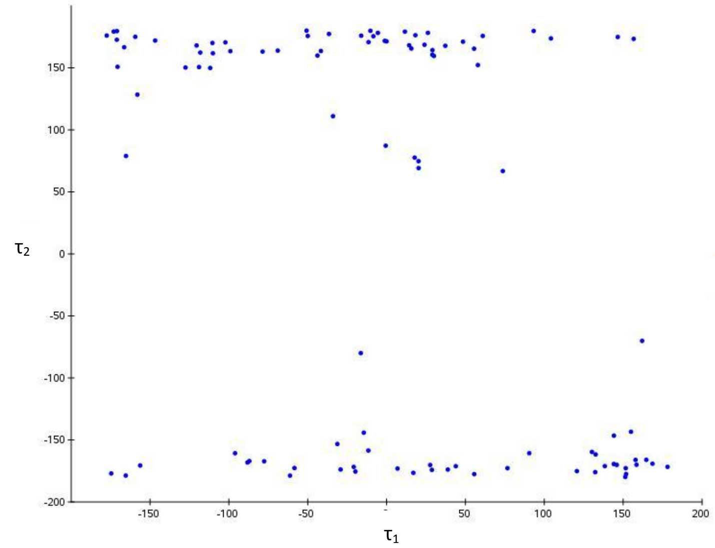

τ_1-τ_2 , *starting conformation: tttg$^+$*

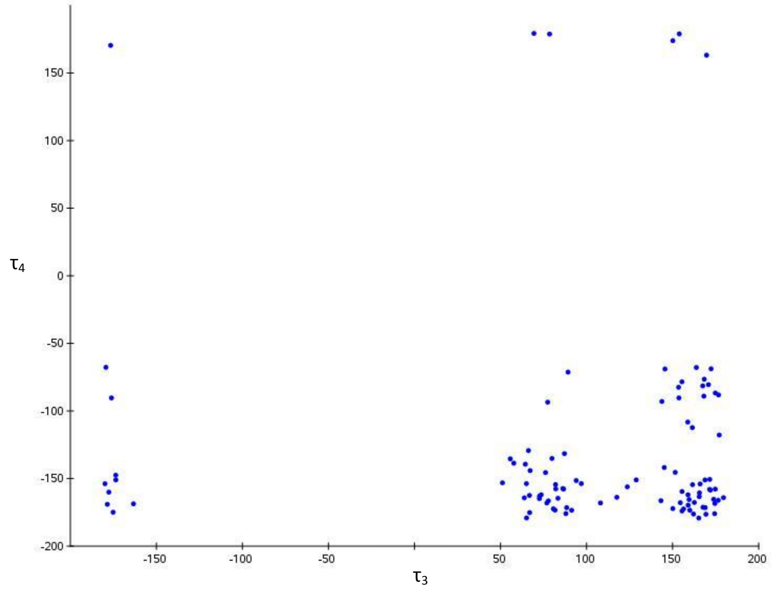

τ_3-τ_4 , *starting conformation: tttg$^+$*

Figure S4

Dihedral angles (τ_1-τ_4) distribution during MD simulations at 300K in vacuum.

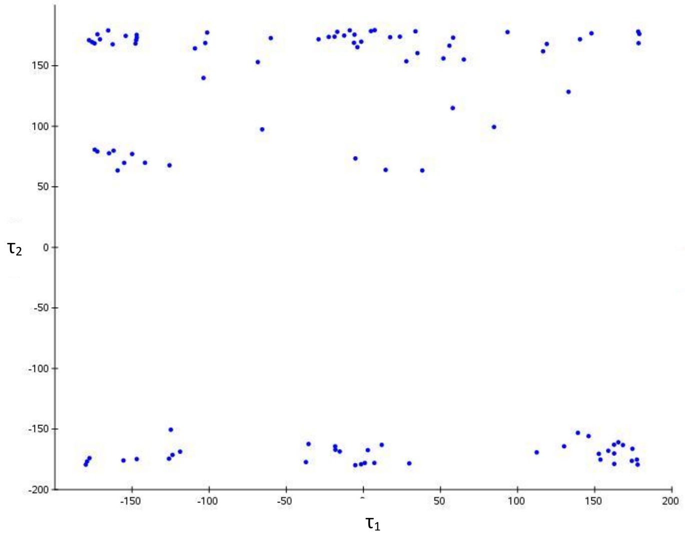

τ_1-τ_2, *starting conformation: tt g^+g^+*

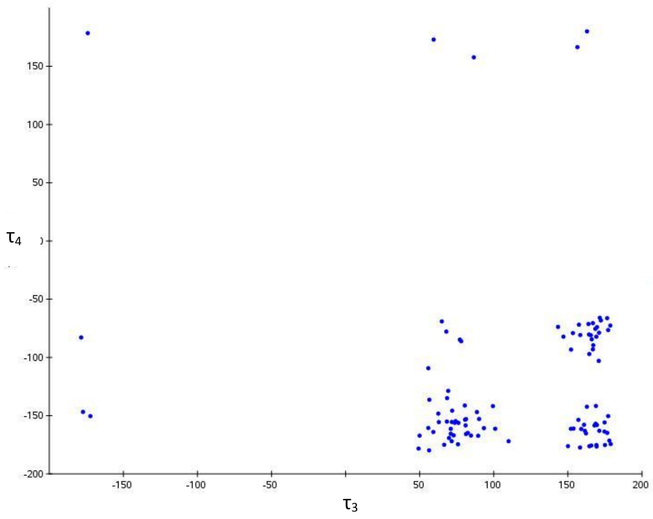

τ_3-τ_4, *starting conformation: tt g^+g^+*

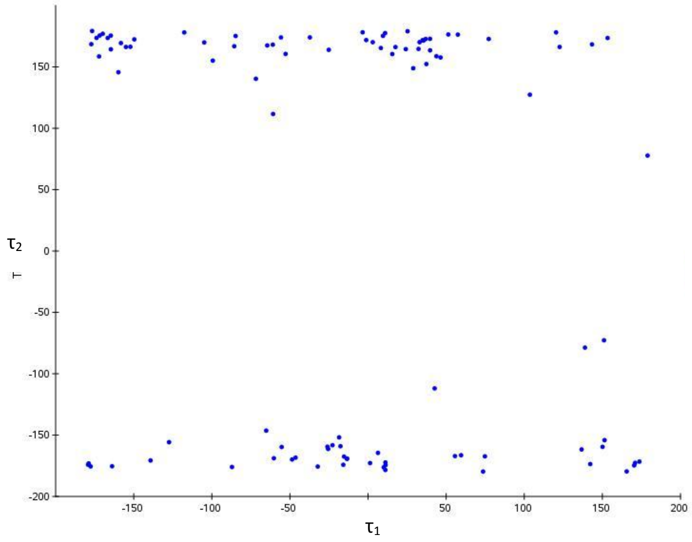

τ_1-τ_2, *starting conformation: tt g^-t*

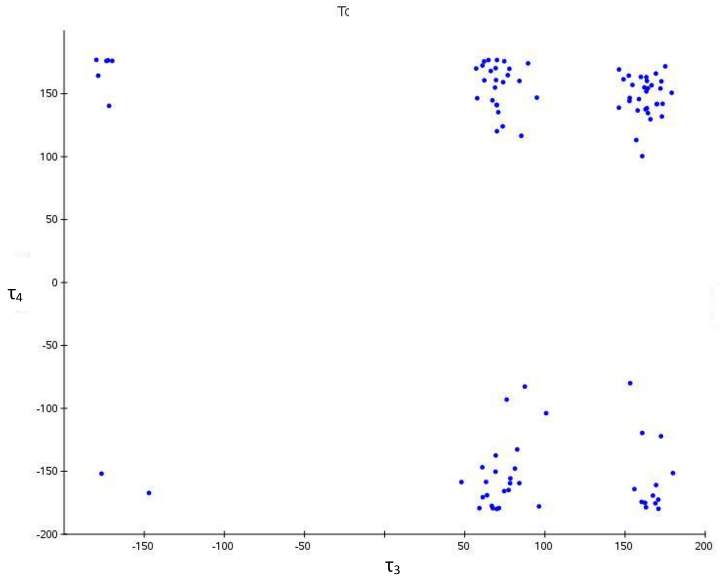

τ_3-τ_4, *starting conformation: tt g^-t*

Figure S5

Dihedral angles (τ_1-τ_4) distribution during MD simulations at 100K in simulated solvent.

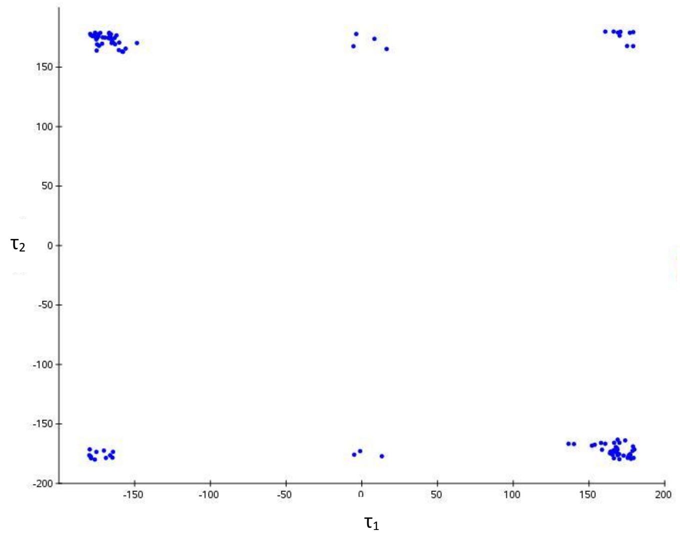

τ_1-τ_2, *starting conformation: all trans*

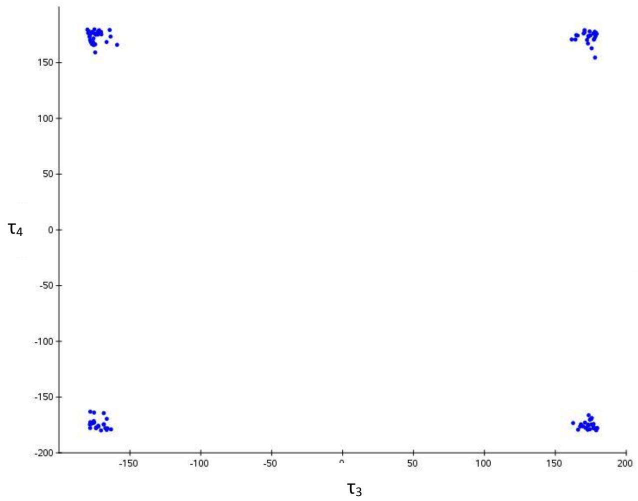

τ_3-τ_4, *starting conformation: all trans*

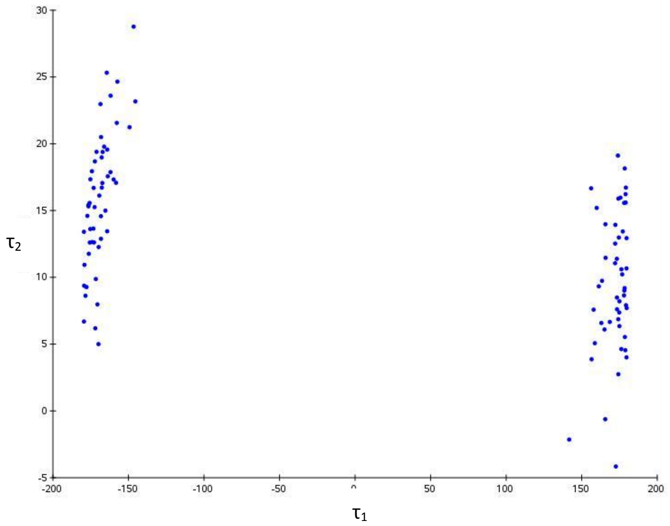

τ_1-τ_2, *starting conformation: tttg$^+$*

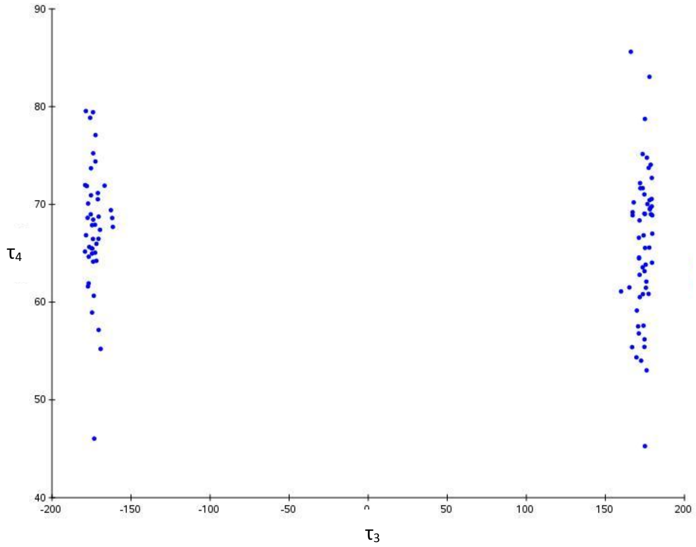

τ_3-τ_4, *starting conformation: tttg$^+$*

Figure S6

Dihedral angles (τ_1-τ_4) distribution during MD simulations at 100K in simulated solvent.

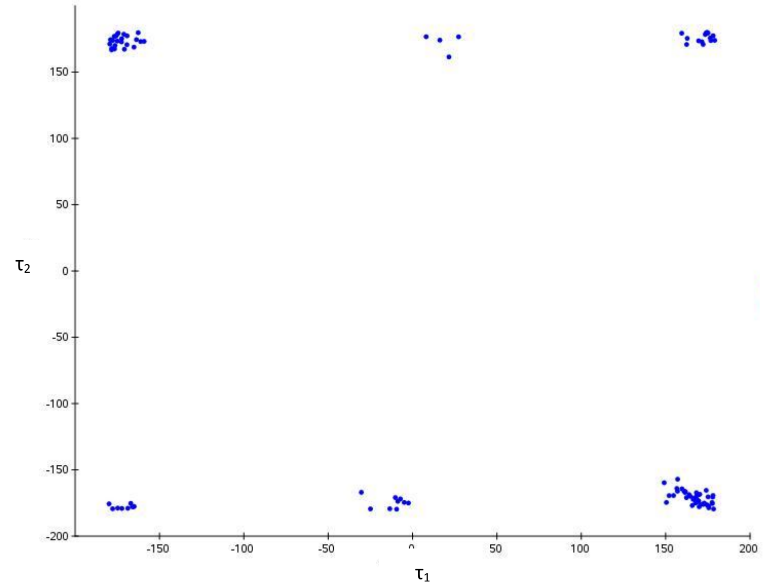

τ_1-τ_2 , *starting conformation: tt g^+g^+*

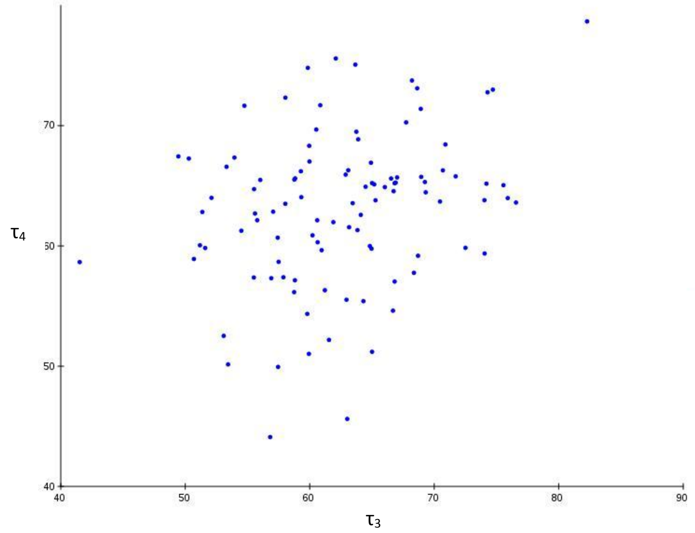

τ_3-τ_4 , *starting conformation: tt g^+g^+*

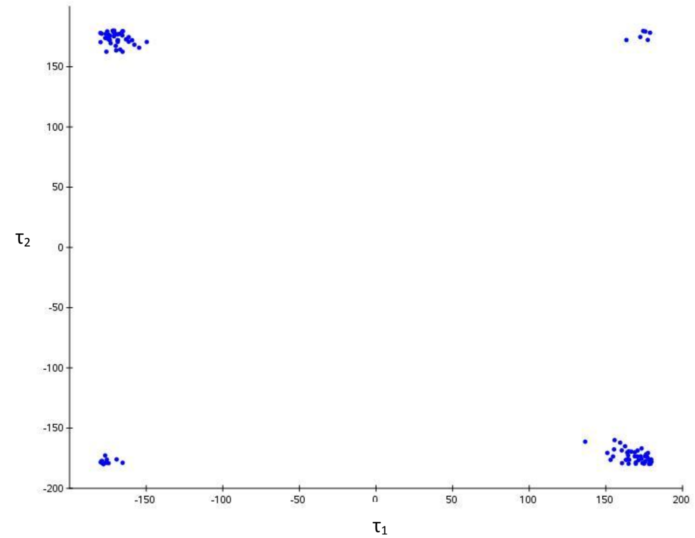

τ_1-τ_2 , *starting conformation: tt g^-t*

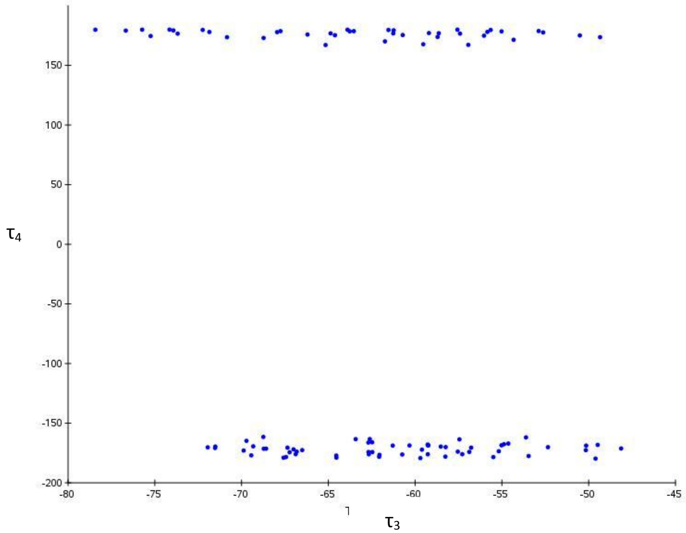

τ_3-τ_4 , *starting conformation: tt g^-t*

Figure S7

Dihedral angles (τ_1-τ_4) distribution during MD simulations at 300K in simulated solvent.

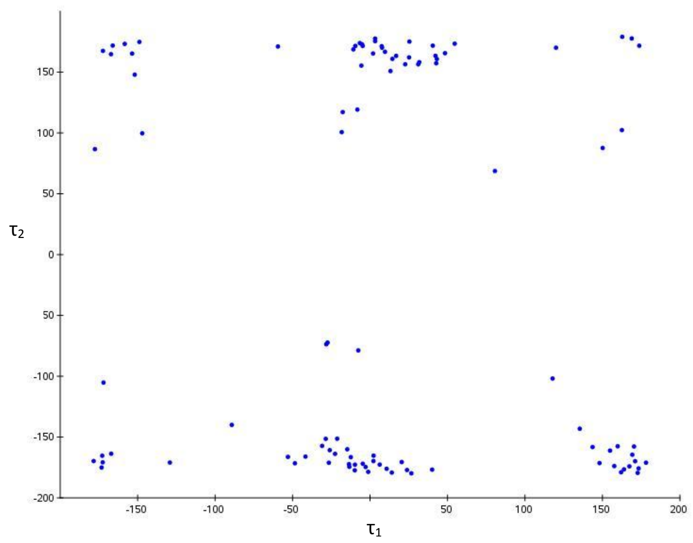

τ_1-τ_2 *, starting conformation: all trans*

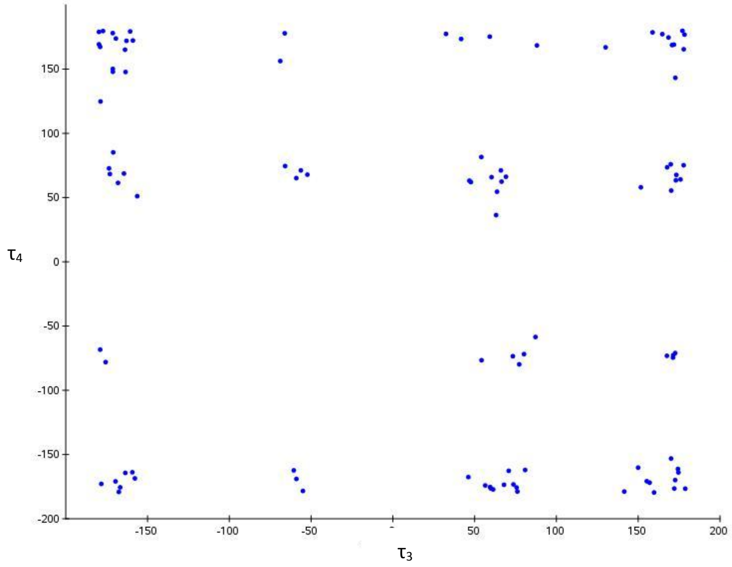

τ_3-τ_4 *, starting conformation: all trans*

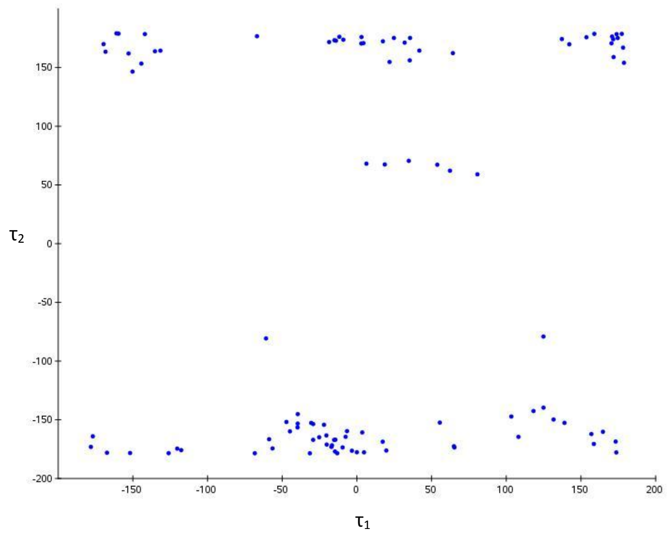

τ_1-τ_2 *, starting conformation: tttg*$^+$

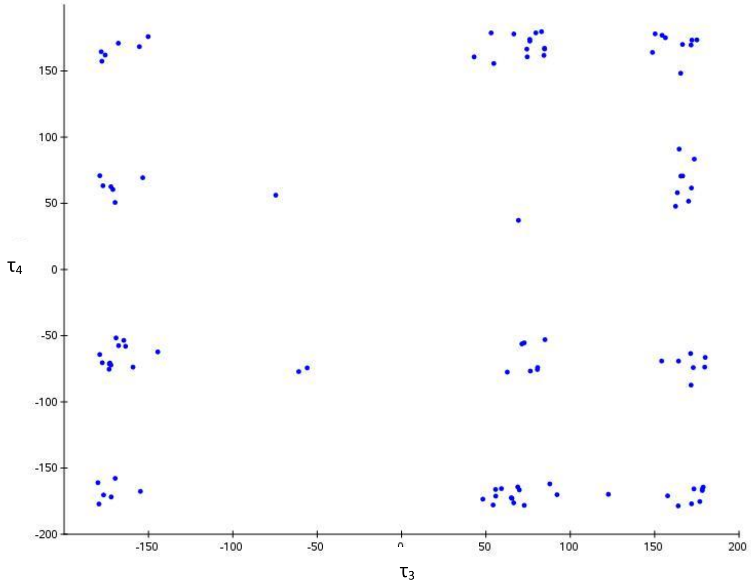

τ_3-τ_4 *, starting conformation: tttg*$^+$

Figure S8

Dihedral angles (τ_1-τ_4) distribution during MD simulations at 300K in simulated solvent.

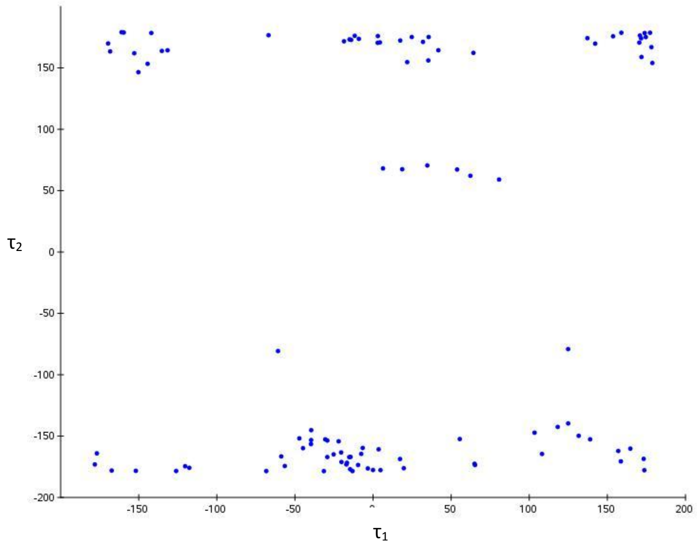

τ_1-τ_2 , *starting conformation: tt g^+g^+*

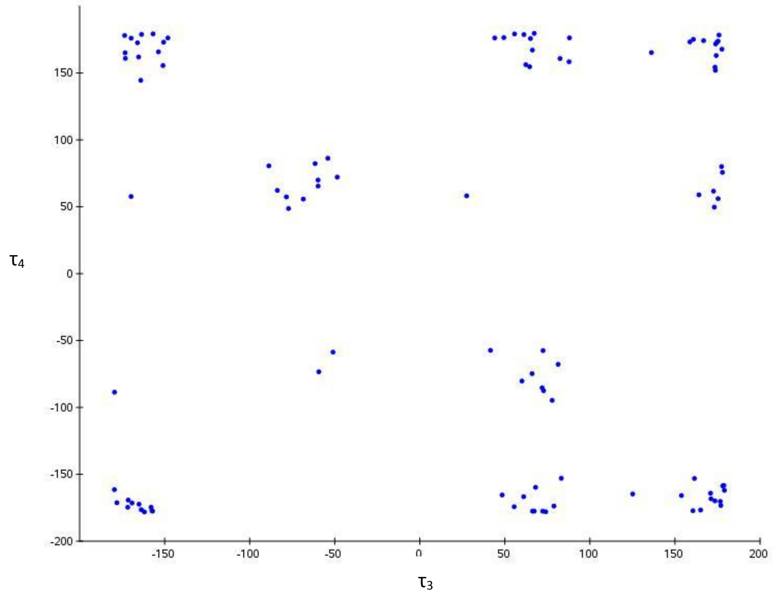

τ_3-τ_4 , *starting conformation: tt g^+g^+*

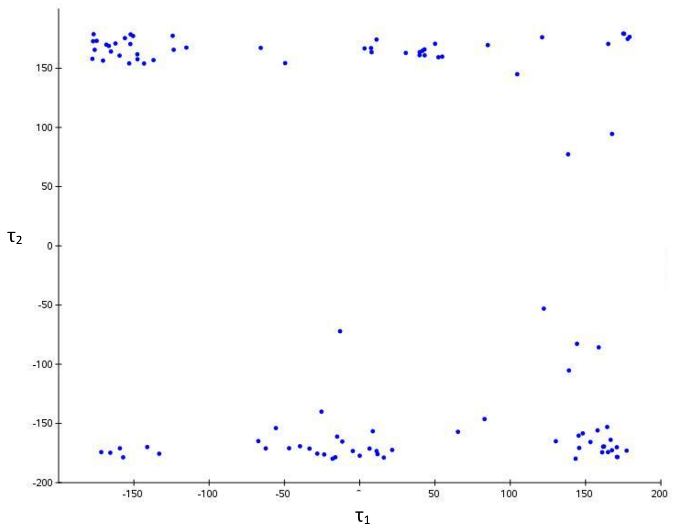

τ_1-τ_2 , *starting conformation: tt g^-t*

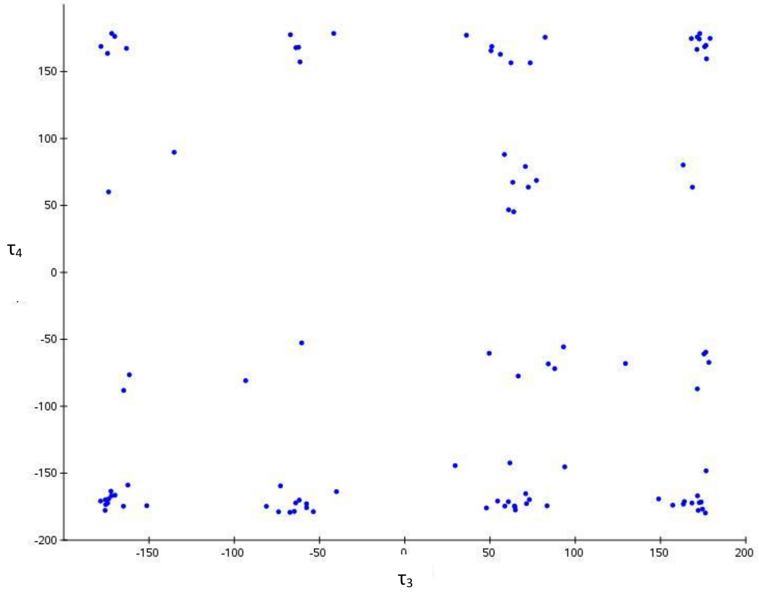

τ_3-τ_4 , *starting conformation: tt g^-t*

Figure S9

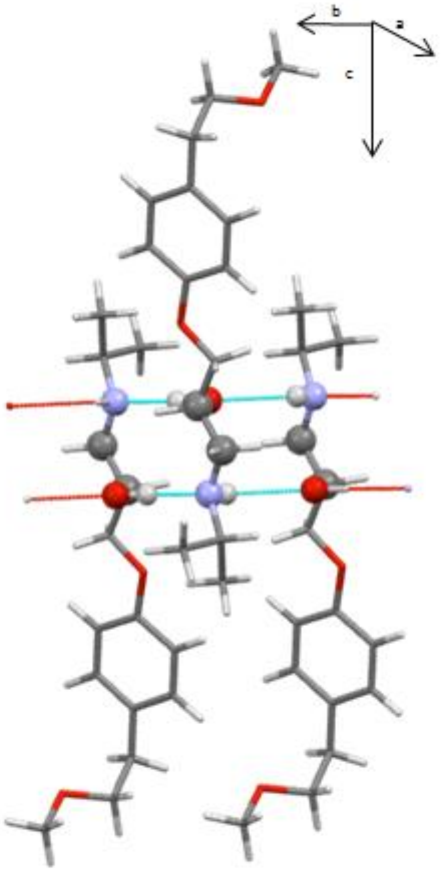

Figure S10: The intra-chain R2,2(10) motifs in **MB** (atoms defining each ring are highlighted)

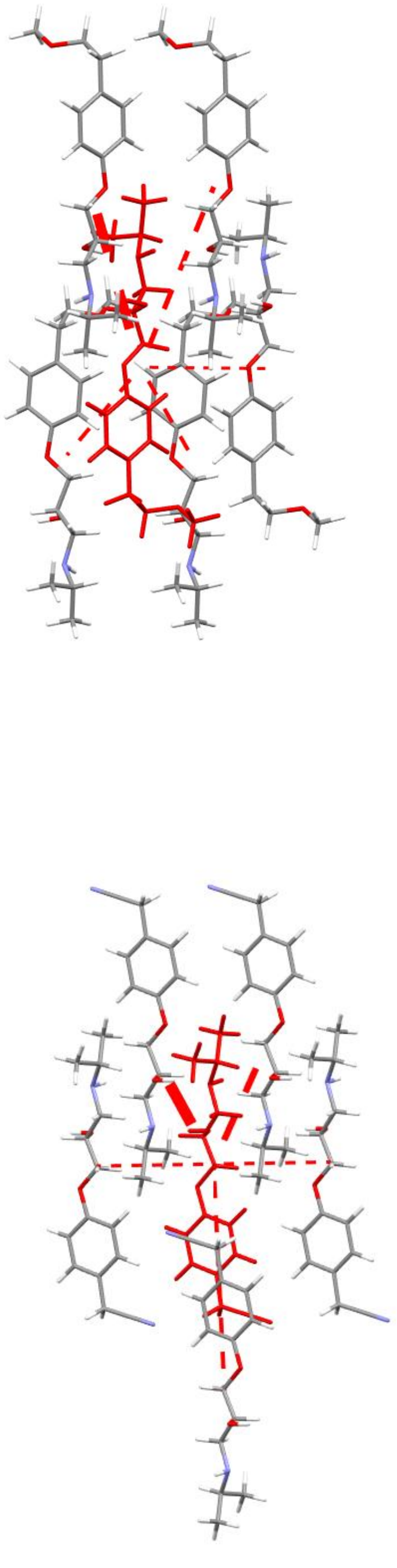

Figure S11: Intermolecular potentials calculated by using the UNI force field (line width set by interaction strength): **MB** (top), **IA** (bottom).

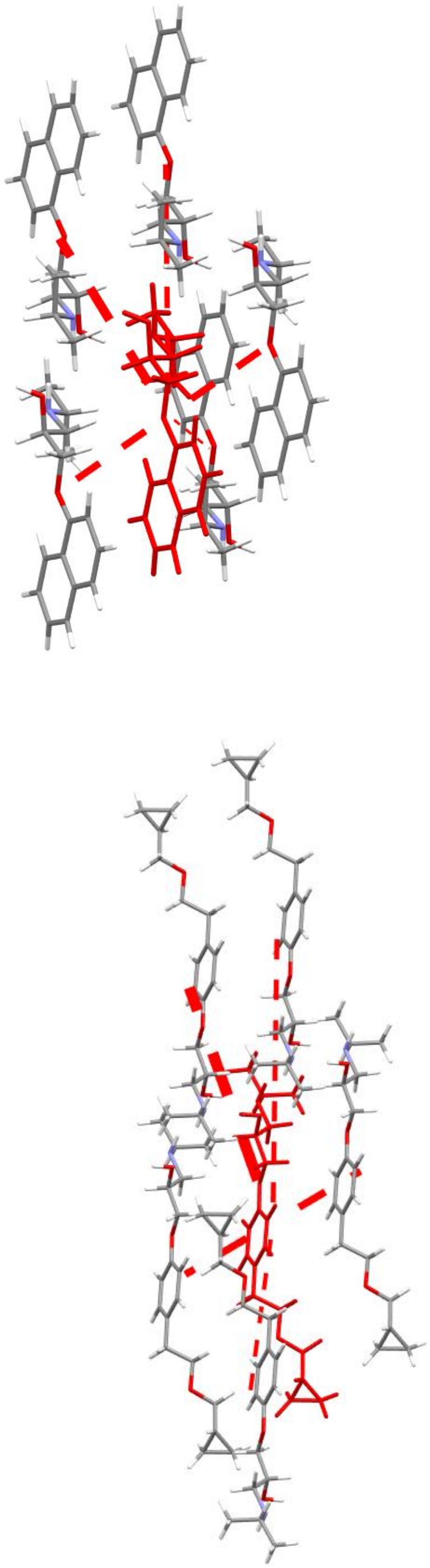

Figure S12: Intermolecular potentials calculated by using the UNI force field (line width set by interaction strength): **PR** (top), **BE** (bottom).

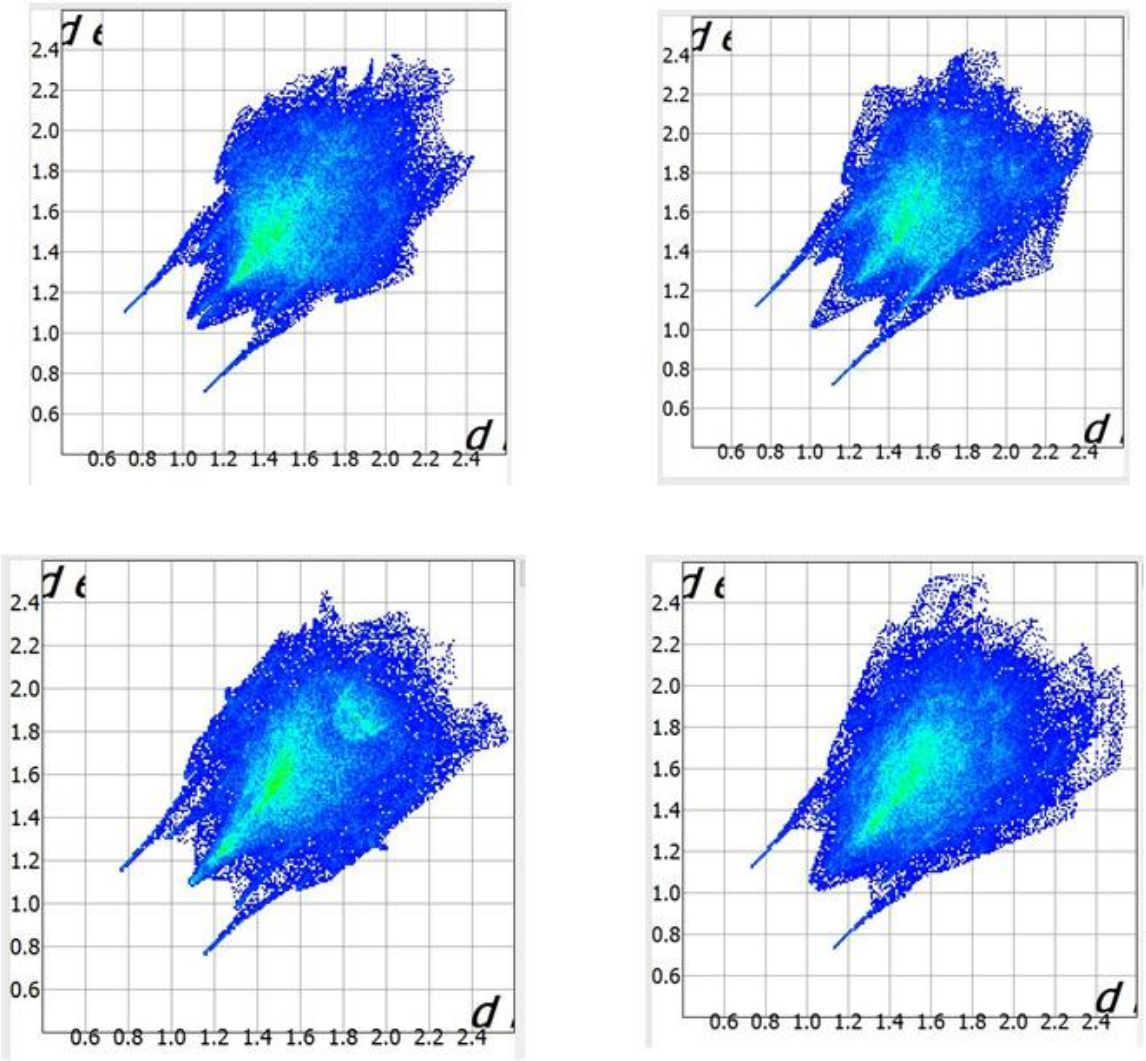

Figure S13: Fingerprint plots for **MB** (up left), **IA** (up right) **PR (**bottom left) and **BE** (bottom right).

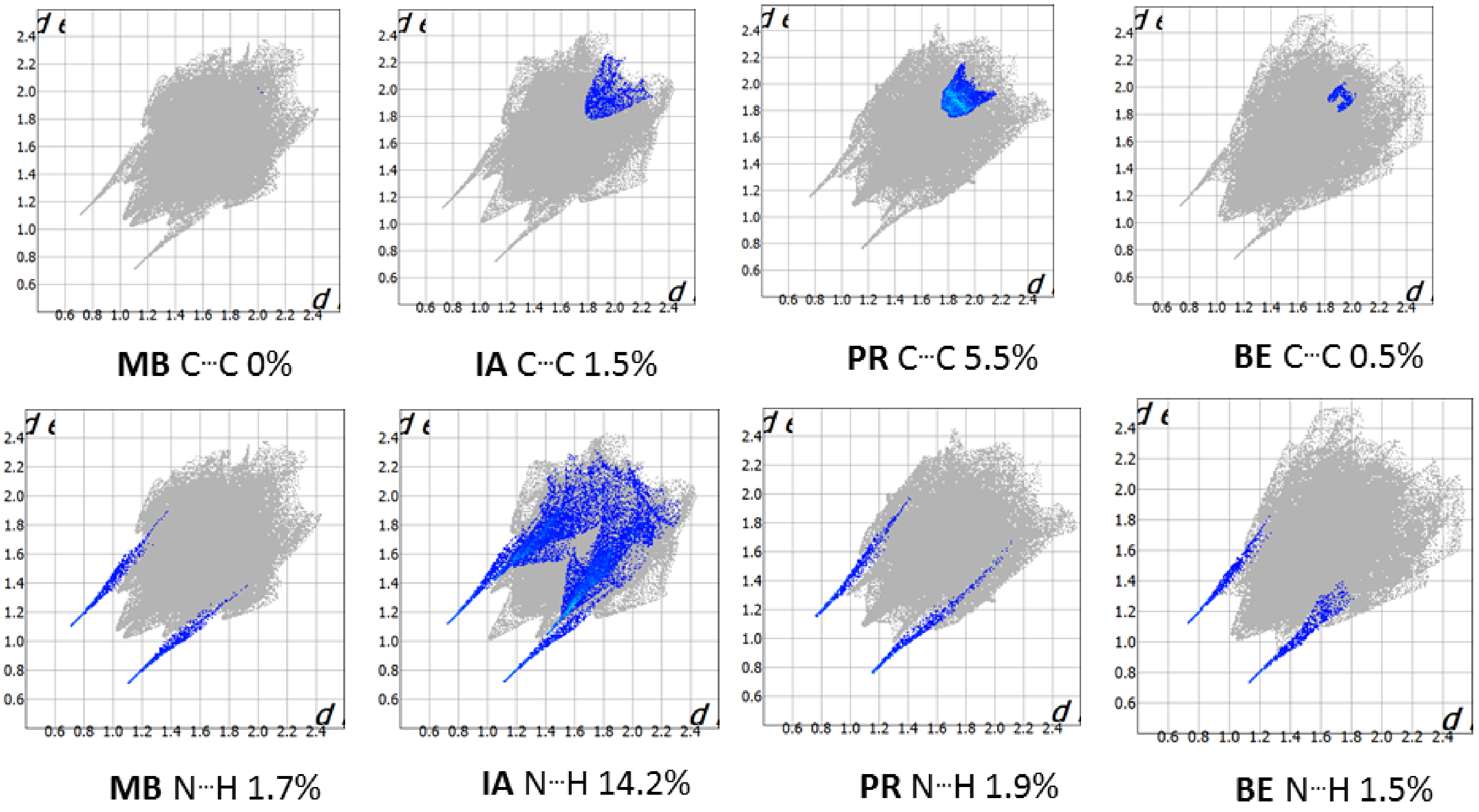

Figure S14: Fingerprint plots for **MB**, **IA PR** and **BE** broken down into contributions from N···H and C···C close contacts (the grey shadow is an outline of the complete fingerprint plot).

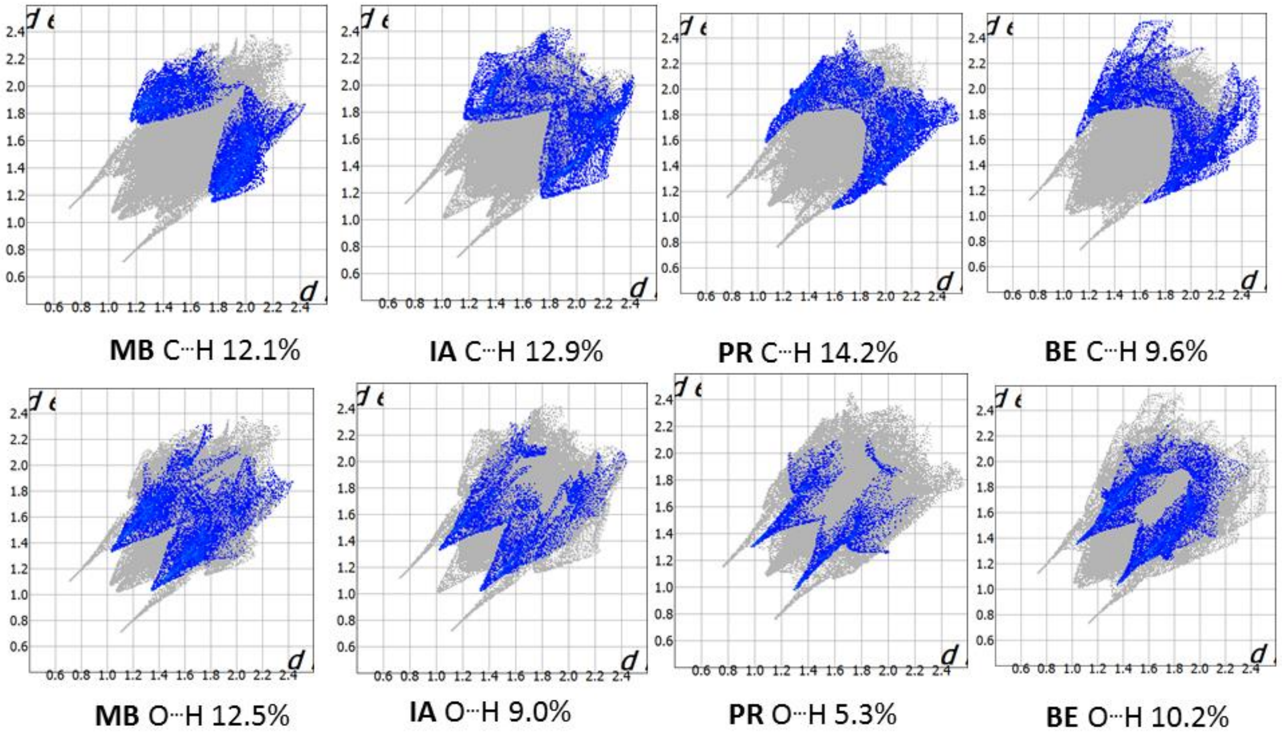

Figure S15: Fingerprint plots for **MB**, **IA PR** and **BE** broken down into contributions from C···H and O···H close contacts (the grey shadow is an outline of the complete fingerprint plot).

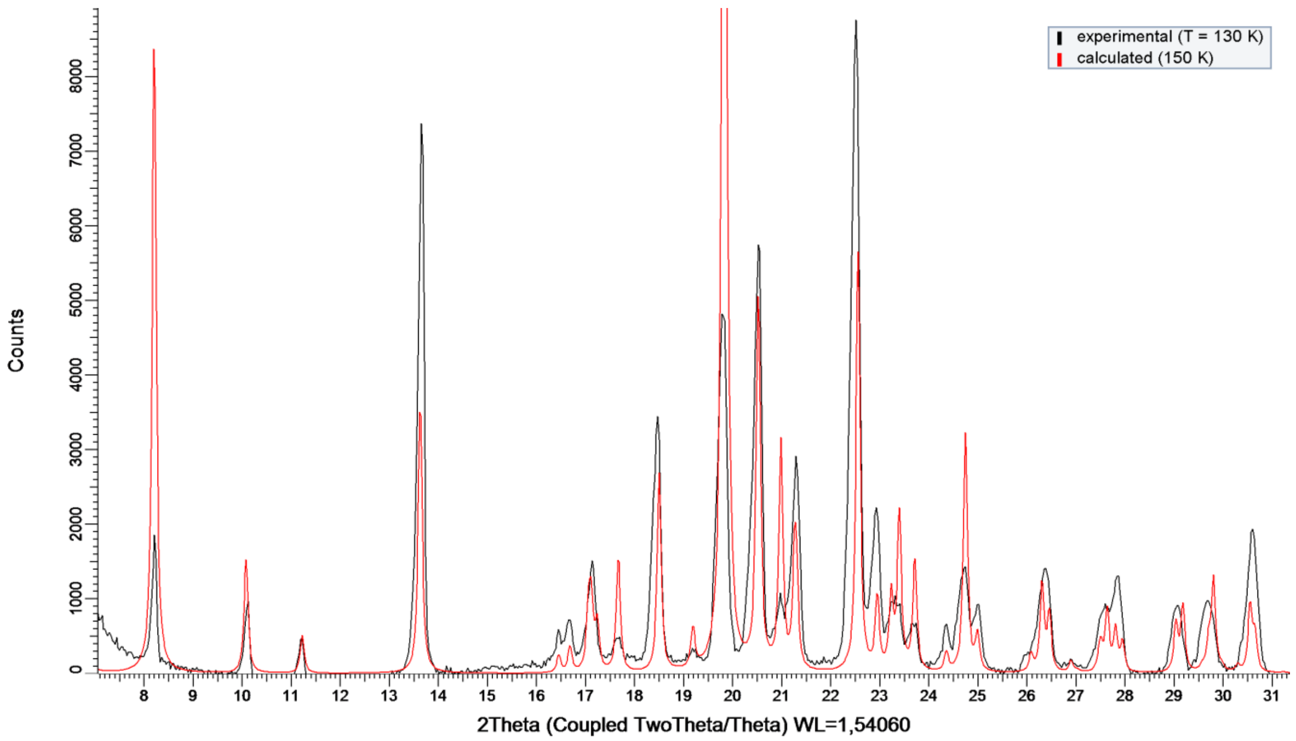

Figure S16: Experimental (130K), calculated (150K) and difference diffraction patterns of **MB**.

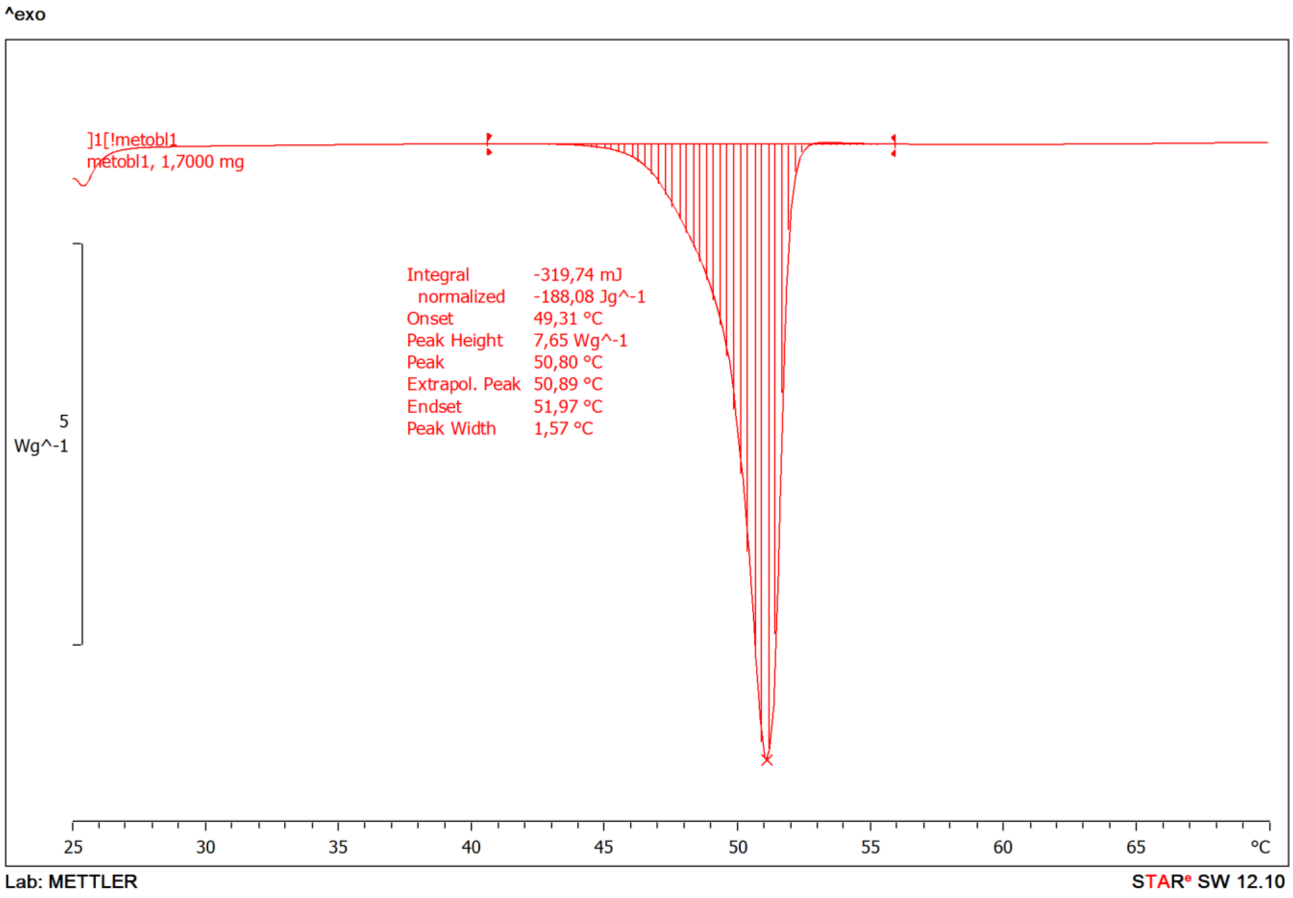

Figure S17: DSC curve of **MB** in the 298-343 K range.

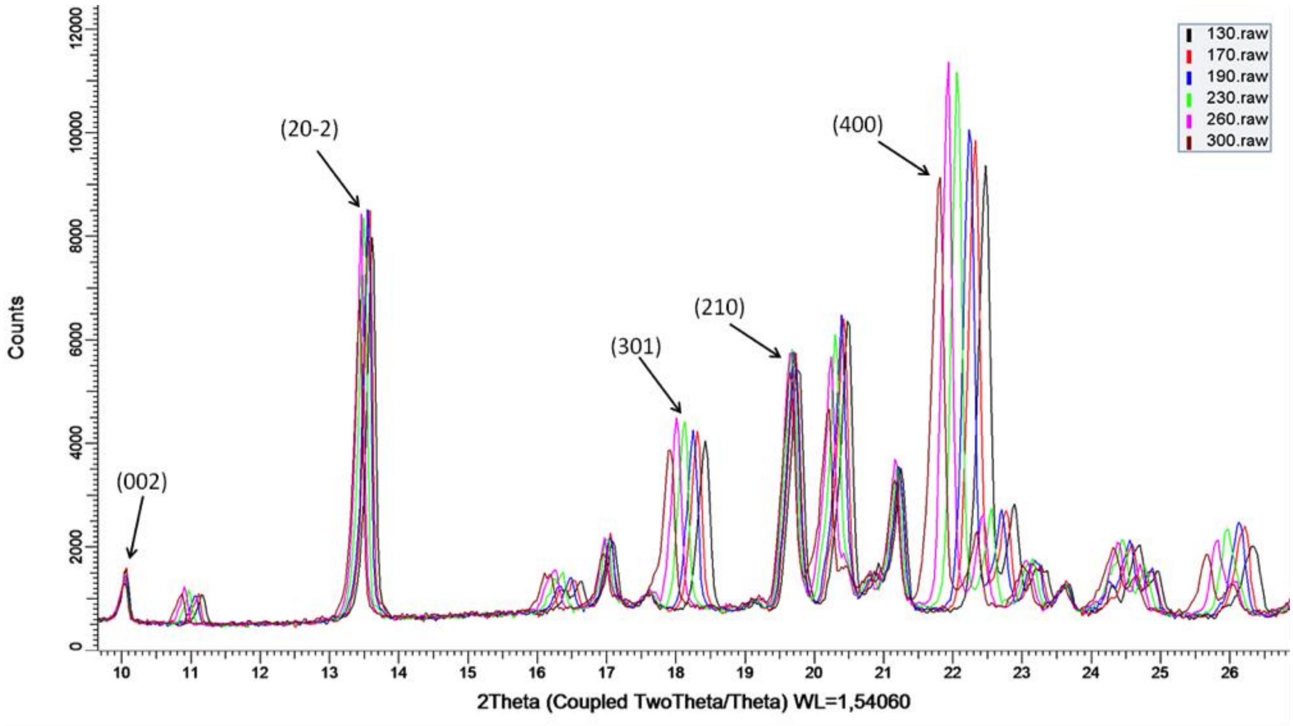

Figure S18: Superimposition of XRPD patterns of **MB** collected in the 130-300 K range.
